# UBTD1: a prognostic and immune biomarker validated in thyroid cancer and pan-cancer analysis

**DOI:** 10.3389/fonc.2026.1850798

**Published:** 2026-07-08

**Authors:** Baoguo Xu, Yue Zhang, Xia Yu, Chenming Guo

**Affiliations:** 1 The First Affiliated Hospital of Xinjiang Medical University, Urumqi, China; 2 Department of Oncology, Bayingol Mongol Autonomous Prefecture People’s Hospital, Korla, China; 3 Department of Dermatology, Xinjiang Uygur Autonomous Region Children’s Hospital, Urumqi, China

**Keywords:** diagnosis, methylation, pan-cancer, prognosis, tumor immunity, UBTD1

## Abstract

**Background:**

Ubiquitin domain containing 1 (UBTD1), a molecule intimately associated with tumorigenesis and progression, is seen as a possible cancer therapy target. However, its diagnostic value, prognostic significance, and immunomodulatory functions of UBTD1 across cancers and in thyroid carcinoma have not yet been fully elucidated.

**Methods:**

Based on TCGA and other public database resources, this research carried out an extensive examination of the expression levels, prognostic potential, diagnostic value, epigenetic modifications, methylation status, immune significance, and pathway regulation of UBTD1, to explore its biological function in diverse malignant tumors. In addition, through *in vitro* experiments such as CCK-8, colony formation, transwell, and oris assays, as well as *in vivo* models of nude mouse xenografts, we validated the expression and regulatory function of UBTD1 in thyroid cancer.

**Results:**

The study indicates that the overexpression of UBTD1 is prevalent in many cancers and correlates with poor prognosis. UBTD1 is effective to a moderate or strong degree in identifying cancerous tissues compared to healthy ones, and it serves as an independent prognostic factor in ACC, LIHC, READ, and THCA patients. UBTD1 mutations are prevalent across various malignancies and correlate with patient prognosis. In the majority of malignancies, UBTD1 expression is positively associated with m6A methylation, showing increased methylation in its promoter region. Moreover, experiments demonstrated that UBTD1 overexpression reduces THCA cell proliferation, invasion, and migration, whereas its under expression exhibited the opposite biological effects.

**Conclusion:**

UBTD1 can be used as a biomarker of important clinical value in pan-cancer. Concurrently, this research clarified the expression and role of UBTD1 in THCA. These findings open new avenues for developing tumor treatments targeting UBTD1.

## Introduction

Cancer is the world’s second leading cause of early death after cardiovascular disease, representing a critical public health priority for the 21st century (1). How to transform cancer from an inevitable health tragedy into a preventable and controllable chronic disease has become a core issue of common concern in the global medical and public health communities. In recent years, the swift advancements in bioinformatics and molecular biology technologies have facilitated the combination of cancer biomarkers with oncology, thereby revolutionizing anticancer therapy and substantially improving both therapeutic outcomes and prognoses for oncologic patients (2). Personalized medicine has ushered in a major transition and a new conceptual framework for cancer diagnosis and management. Based on the specific molecular features of an individual’s tumor, clinicians can employ biomarkers to design accurate and personalized cancer therapies (3).

Ubiquitination is a crucial post-translational modification involved in protein degradation and regulation, essential for processes like DNA repair, cell cycle control, and immune response (4). UBTD1 is a protein that contains a domain similar to ubiquitin, characterized by high evolutionary conservation across diverse species. Besides, it has been reported to interact with particular E2 and E3 enzymes that participate in the ubiquitin−proteasome system across cellular and animal models (5). Recent investigations have continuously verified the implication of UBTD1 in the development and advancement of various malignancies. According to Zhang (6), UBTD1 exerted a senescence-inducing effect in gastric cancer cells through enhancing p53 stability and facilitating Mdm2 degradation. Torrino (7) demonstrated that UBTD1 depletion profoundly modulated the mechanical features of epithelial cancer cells and greatly promoted invasive capacity by activating RhoA. Earlier studies demonstrated that increased UBTD1 expression promotes senescence in both human fibroblasts and malignant cells, consequently lowering their tumor-initiating potential (8). In lung cancer, prostate cancer, and hepatocellular carcinoma, the above studies demonstrated that UBTD1 catalyzes the degradation of YES1-related transcriptional regulator (YAP) and inhibits the progression of lung, prostate, and liver cancers (7, 8). Previous research has demonstrated the importance of UBTD1 in cancer, it acts as a tumor suppressor in some cancers and as an oncogene in others. however, comprehensive analyses encompassing all cancer types remain lacking. The objective of this research was to investigate the connection between UBTD1 and both the prognosis and immunotherapy outcomes in human cancers.

This investigation focused on understanding the involvement of UBTD1 in cancer formation by analyzing its expression in 33 different cancer types through various databases. Furthermore, the research evaluated the significance of UBTD1 in prognosis and diagnosis, as well as its relationship with immune cell infiltration. Functional enrichment analysis was also conducted. Notably, the study focused on UBTD1’s role in thyroid cancer using cell and animal experiments, laying the groundwork for future research. The results enhance the understanding of UBTD1’s role in cancer, suggesting its influence on patient prognosis and potential as a treatment target.

## Methods

### Data sources and preprocessing

The research leveraged the UCSC XENA database (9) (https://xenabrowser.net/datapages/) to access RNA sequencing data and clinical details from TCGA for carcinoma, alongside expression in normal tissue samples sourced from the GTEx database. A dataset was created, encompassing 33 cancer types and 15,776 samples, including 9,807 TCGA tumor samples, 727 adjacent non-tumor tissues, and 7,568 GTEx normal tissues. The expression profile data were converted into a log2-transformed TPM format to facilitate subsequent analysis.

### UBTD1 expression analysis

The analysis of mRNA expression differences of UBTD1 was conducted using the “gplot2” and “rstatix” packages across the TCGA dataset, the TCGA-GTEx integrated dataset, and paired samples. Simultaneously, the HPA database (10) (https://www.proteinatlas.org) was leveraged for the analysis of immunohistochemical (IHC) data from 13 types of malignant tumors, aiming to confirm protein-level differences in UBTD1 expression.

### Analysis of UBTD1’s prognostic and diagnostic significance in pan-cancer

Use the “survival”, “survminer” package for single variable and Cox regression analysis with the help of Kaplan Meier - Plotter database (11) (https://kmplot.com/analysis/) analysis, the relationship between UBTD1 expression and clinical prognostic outcomes, with a focus on OS, was confirmed. Subsequently, the “ggplot2” package was utilized to create a forest plot for the visualization of the findings.

The ROC curve was analyzed using the “pROC” in the R statistical environment, and “ggplot2” was used for visualization. The AUC was used to evaluate UBTD1’s diagnostic effectiveness: scores of 0.7-0.9 imply moderate discriminative performance, whereas scores greater than 0.9 signify excellent performance.

### Development and calibration of prognostic nomograms

Researchers utilized Cox regression analysis to pinpoint potential prognostic factors for patients. The division of samples was based on UBTD1 expression, resulting in high and low subgroups, serving as an independent prognostic factor. Factors showing any variable with a P-value<0.1 in univariate analysis were then incorporated into the multivariate model to screen for significant prognostic indicators. These were then used in a prognostic nomogram, constructed and visualized with the “rms” package in R. A C-index obtained from 1,000 replicates was used to assess the predictive precision of the nomogram. Additionally, a calibration curve was developed to assess the concordance between the predicted survival outcomes and the empirically observed results.

### Genetic variation and methylation analysis of UBTD1

With the help of the Oncoprint tool on the cBioPortal database (12) (https://www.cbioportal.org/), genetic variations of UBTD1 in the TCGA Pan-Cancer Atlas were detected. The “Cancer Types Summary module” assessed UBTD1 gene mutations, mutation types, and copy number variations (CNV) recurrence across cancer types. The “Mutation” module assessed UBTD1 mutation sites. Using the “Mutation-CNV” function in the GSCA (13, 14) database (https://guolab.wchscu.cn/GSCA/#/), we explored the association of UBTD1 CNV with its mRNA expression and clinical prognosis in pan-cancer datasets.

We analyzed the methylation profile of the UBTD1 promoter in cancer and adjacent normal tissues using the UALCAN database (15) (https://ualcan.path.uab.edu/) and by analyzing the correlation between UBTD1 mRNA expression and 24 core m6A methylation regulatory factors using the Sangerbox 3.0 (16) online database (http://vip.sangerbox.com/).

### Association analysis between UBTD1 and drug sensitivity

The present study sought to characterize the function of UBTD1 in modulating the drug response of tumor cells by utilizing the GSCA database to investigate the influence of UBTD1 mRNA levels on drug susceptibility. The GSCA platform amalgamates data from the GDSC and the CTRP, encompassing a total of 750 small molecule compounds. In this research, a positive correlation means higher expression levels of UBTD1 predict reduced drug sensitivity, while an inverse correlation suggests it may increase drug sensitivity.

### Tumor Immune-Related Analysis of UBTD1

The link between UBTD1 and different tumor traits, including tumor mutational burden (TMB), microsatellite instability (MSI), neoantigen load (NEO), MATH score, homologous recombination deficiency (HRD), and loss of heterozygosity (LOH), was analyzed using the Sangerbox 3.0 database. Results were visualized with a radar chart via the R package “ggplot2”. Analyses were conducted with the “estimate” package to determine the tumor microenvironment (TME) through StromalScore, ImmuneScore, and ESTIMATEScore. To explore UBTD1’s relationship with immune-related genes, a gene list from the GSEA database (17) (https://www.gsea-msigdb.org/gsea/msigdb/index.jsp) was used for correlational assessment using the Spearman coefficient.

The immune cell infiltration profiles of 24 different immune cell types were analyzed utilizing the GSVA package (17, 18). Combined with the TIMER3.0 database (19) (http://timer.cistrome.org/), the EPIC, MCP-COUNTER, CIBERSORT, and TIDE algorithms were used to assess how UBTD1 expression correlates with immune cell infiltration levels, including CD8+ T cells and cancer-associated fibroblasts (CAFs).

### Profiling of the PPI network and enrichment analysis of functional annotations

Utilizing the STRING database (20) (https://cn.string-db.org/, v11.5), we constructed the protein-protein interaction (PPI) network for UBTD1, and 50 interacting proteins were screened. The results were visualized with Cytoscape software (21). Subsequently, genes correlated with UBTD1 were recognized by means of the GEPIA3 database (22) (https://gepia3.bioinfoliu.com/), followed by cross-validation against interacting proteins in the STRING database. Venn diagrams were used to extract overlapping targets. Functional annotation analysis using GO and KEGG pathways was conducted with the “clusterProfiler” and “GOplot” packages in R. Initially, the input molecular list was converted to gene IDs, followed by enrichment analysis with the “clusterProfiler”. Screening criteria were defined by a Benjamini-Hochberg adjusted P-value of less than 0.05 and a minimum gene set size of at least 10. The outcomes of these analyses were subsequently visualized. Only the top 5 entries with the highest tumor relevance for each category were displayed in the resulting plots.

For the purpose of examining UBTD1’s functional pathways in multiple malignancies, TCGA samples were separated into high and low expression categories using the median UBTD1 expression as a benchmark. Differential gene expression analysis was performed with the help of “DESeq2” and “edgeR”, followed by GSEA with “clusterProfiler”. The reference gene set was obtained from “c2.cp.all.v2022.1.Hs.symbols.gmt” in the MSigDB database (23) (https://www.gsea-msigdb.org/gsea/index.jsp), with 5000 permutations. Ridge plots were employed to visually represent the top ten “Reactome” pathways associated with each cancer type.

### Tissue collection

Sixteen pairs of clinical thyroid cancer tissues and matched adjacent normal tissues were obtained from patients receiving radical thyroidectomy at the First Affiliated Hospital of Xinjiang Medical University between August 2023 and May 2024. Inclusion criteria included confirmed THCA via postoperative pathology and complete medical records. Exclusion criteria included non-primary THCA and prior radiotherapy, chemotherapy, or hormone therapy. Approval for this study was granted by the hospital Ethics Committee (Approval No.: 240714-09), and informed consent was acquired from all subjects before their recruitment.

### Cell culture

THCA cell lines B-CPAP (CL-0575, Procell, China) and TPC-1 (CL-0643, Procell, China) were cultured in specific medium (CM-0575 for B-CPAP; CM-0643 for TPC-1, Procell, China) in an incubator under conditions of 37°C, 5% CO_2_ and saturated humidity. Subculture was performed when cell confluence reached 80%–90%.

### Lentiviral packaging and concentration

Three shRNA sequences targeting UBTD1 (**Supplementary Table 1**) were constructed, subcloned into the MCS region of the lentiviral vector GV493, and verified by enzymatic digestion and sequencing. Meanwhile, the target fragment was amplified from the plasmid containing the full-length UBTD1 cDNA and ligated into the GV385 overexpression vector driven by a specific promoter; successful construction was confirmed by sequencing. For virus production, 2 μg of verified interference or overexpression plasmid plus packaging plasmids were co-transfected into well-growing HEK-293T cells (transfection efficiency ≈80%) using Lipofectamine 3000 (L3000015, Thermo Fisher Scientific, China) following the manufacturer’s instructions. At 48 hours following transfection, the supernatants were harvested, and lentiviruses were concentrated by ultracentrifugation. The precipitate was resuspended in a dedicated buffer and preserved at -80°C.

### Lentiviral infection and grouping

When B-CPAP and TPC-1 cells reached 80% confluence, lentiviral suspension was added at an MOI of 8. After 24 h of incubation, the medium in the culture was swapped out, and the cells continued to incubate for 48 hours. Four experimental groups of cells were established: NC-KD (UBTD1 knockdown control), KD (UBTD1 knockdown), NC-OE (UBTD1 overexpression control), and OE (UBTD1 overexpression). Their status was examined under a fluorescence microscope 72 hours after infection.

### Western blot

RIPA lysis buffer containing PMSF protease inhibitor (Beyotime, Shanghai, China) was applied to extract proteins from both tissue and cell samples, followed by quantitative analysis using a BCA protein assay kit. Following separation by SDS-PAGE and transfer to membranes, incubation of the membranes was performed overnight at 4°C with an anti-UBTD1 antibody (1:1000; 20158-1-AP, Proteintech, China). Subsequent to incubation, the membranes were treated with an HRP-conjugated secondary antibody at a dilution of 1:5000 (RGAR001, Proteintech, China) for a duration of 1.5 hours at ambient temperature. Visualization of the protein bands was achieved using an enhanced chemiluminescence (ECL) kit (Biosharp, Beyotime, China) and captured using a Bio-Rad imaging system. Membranes were stripped with stripping buffer (sw3022, Solarbio, China), then re-probed with anti-GAPDH antibody (ab181602, Abcam, USA) as the loading control.

Membranes from B-CPAP and TPC-1 cells underwent overnight incubation at 4°C with anti-UBTD1 and anti-β-actin antibodies (both 1:1000, Proteintech, China). After applying species-specific secondary antibodies (1:5000, Proteintech, China), the bands were detected and recorded.

### Assessment of cell proliferation using CCK-8 assay

Cell proliferation was assessed utilizing a CCK-8 assay kit (Dojindo Molecular Technologies, Japan). Cells were seeded into 96-well plates and cultured over a period of 1 to 5 days. CCK-8 reagent was introduced into each well at predetermined times, followed by a 1.5-hour incubation at 37 °C. Absorbance was then measured at 450 nm with a microplate reader. A line graph was generated to illustrate variations in proliferative capacity over time.

### Colony formation assay for cell proliferation

B-CPAP and TPC-1 cells were seeded into 6-well plates at a concentration of 800 cells per well and maintained for 14 days. After discarding the culture medium, the cells were washed with phosphate-buffered saline (PBS) and then fixed with 4% paraformaldehyde for 20 minutes, and washed again. Following this, a 0.1% crystal violet solution was used to stain the cells for 20 minutes, followed by rinsing and air-drying. An inverted microscope at 200× magnification was employed to observe and record the colonies. The colony formation rate was determined using ImageJ 6.0 software, evaluated as (number of colonies/number of seeded cells) × 100%.

### Oris cell migration assay

Oris™ cell migration stoppers were disinfected with alcohol, dried, and placed into 96-well plates. Infected cells were seeded according to groups to reach ≈90% confluence the next day. Oris™ stoppers were carefully removed, and plates were scanned for baseline imaging before further incubation. Image acquisition was performed at both 0 h and 48 h time points. Cell migration was evaluated by quantifying the change in the cell-free area. To find the migration rate, the following formula was applied: Cell migration rate (%) = [(initial blank area at 0 hours − blank area at 48 hours)/initial blank area at 0 hours] × 100%.

### Transwell assay for cell migration and invasion

Transwell assays (Corning, NY, USA) were employed to determine cell migratory and invasive capacities. After treatment, B-CPAP and TPC-1 cells were plated into the upper compartments at a density of 1×10^5^ cells per well in 200 μL serum-free medium. The lower compartments were filled with 600 μL culture medium containing 10% FBS. Following 24 hours of incubation, the cells adhering to the upper chamber were gently wiped away. The invaded cells were then fixed with 4% paraformaldehyde for a duration of 30 minutes, stained with 0.1% crystal violet for 15 minutes (Beyotime, Shanghai, China), rinsed with PBS, and allowed to air dry. Cells were observed and imaged at 200× magnification using a Leica inverted microscope, and counted in five random fields.

### Animal experiments

All animal experimental procedures were evaluated and sanctioned by the Animal Ethics Committee of the First Affiliated Hospital of Xinjiang Medical University (Approval No.: A240801-184). Fifteen NCG mice, each six weeks old, were procured from Beijing Vital River Laboratory Animal Technology Co., Ltd. (Animal Quality Certificate No.: SCXK (Beijing) 2021-0006). The fifteen NCG mice were randomly allocated into three groups using R (version 4.4.2), comprising five mice per group. Five mice per group were selected for statistical, literature and ethical reasons. First, n=5 retains enough replicates for Student’s t-test and one-way ANOVA if one mouse develops abnormal tumors or is lost; groups of ≤3 mice cannot support valid statistics after removing outliers. Second, this sample size matches standard values in relevant published tumor mouse studies for cross-study comparison. Third, the design adheres to the 3R principle to minimize animal numbers without losing statistical reliability. Furthermore, all measurements, including tumor volume, body weight, and subsequent molecular analyses, were performed by researchers who were blinded to the group allocations to ensure unbiased assessment. TPC-1 cells were cultured to over 80% confluence and infected with lentivirus, then divided into three groups: UBTD1 negative control (NC), knockdown (KD), and overexpression (OE). After digestion and counting, cells were resuspended in cold PBS, mixed with Matrigel at a 1:1 ratio, and prepared at 5×10^6^ cells/100 μL for subcutaneous injection into mice. Every three days, measurements of the tumor’s dimensions were taken beginning on day 7 following inoculation, and tumor volume was computed according to the equation V = 0.5×L×W². Mice were monitored daily for general health, including body weight, food and water intake, activity, and paw pad and lip color. Particular emphasis was directed toward integrity of the local skin over the tumor, checking for redness, swelling, ulceration, bleeding, or purulent discharge. Tumor texture and adhesion to surrounding tissues were assessed by palpation. Sepsis screening was performed immediately if the body temperature of mice exceeded 40 °C accompanied by listlessness. Euthanasia was performed when the average tumor volume of the control group approached 1,000 mm³, or any mouse in the experimental groups reached the preset humane endpoints. Humane endpoints included: subcutaneous tumor volume ≥2000 mm³ (or diameter ≥2 cm), or tumor weight accounting for 10%–15% of body weight; tumor ulceration, bleeding, pyogenic infection, or limb motor dysfunction caused by tumor invasion into joints and muscles. Euthanasia was performed by intraperitoneal injection of an excessive dose of 5% sterile pentobarbital sodium solution (P3761, Merck, Germany) at 150 mg/kg. Before operation, mice were properly fixed and the abdominal skin was disinfected. The needle was inserted at an angle of 45° to a depth of 5–8 mm. After confirming no blood or intestinal fluid by aspiration, the solution was slowly injected within 10 seconds, and the mice were observed continuously for at least 5 minutes. The criteria for death were muscular relaxation of limbs, disappearance of corneal reflex, and cessation of respiration and heartbeat. Subsequent processing was performed only after death was confirmed. All operators had received at least 10 standardized practical training sessions and passed the assessment. Finally, tumors were dissected, weighed, and used for subsequent molecular biological analyses.

### Statistical analysis

R (version 4.4.2) was employed for statistical analyses, while figures were plotted with the “ggplot2” package. The specimens were classified into high− and low−expression groups using the median UBTD1 mRNA level as the optimal threshold. To assess differences in UBTD1 expression levels, unpaired samples were analyzed using the Mann-Whitney U test, and paired samples were assessed with the Wilcoxon signed-rank test. Kaplan−Meier curves were constructed for survival analysis and compared via the log−rank test. A P-value below 0.05 was considered statistically significant.

## Results

### UBTD1 expression landscape across cancer types

**Figure 1:**
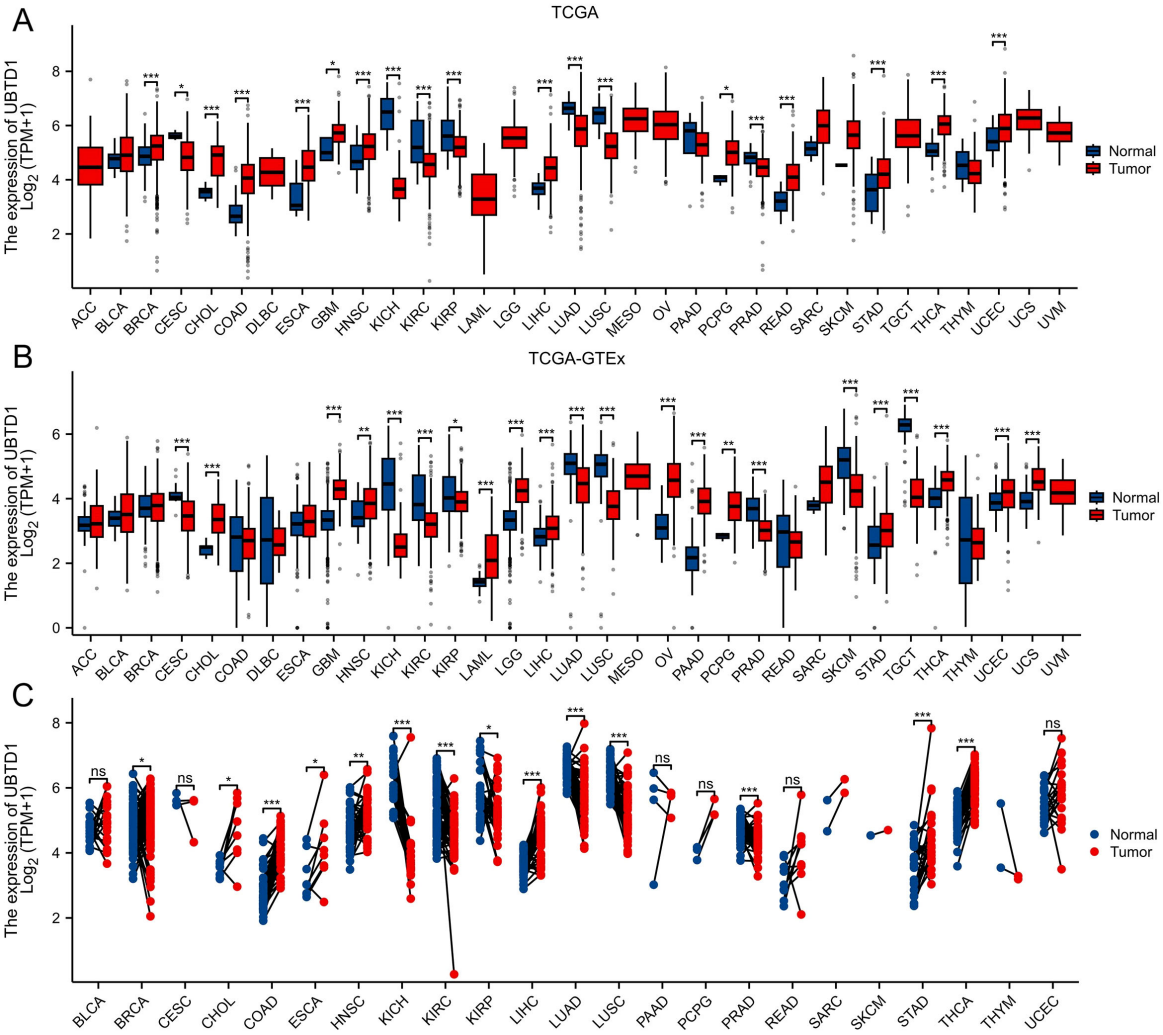
Analysis of UBTD1 mRNA expression across 33 cancer types: **(A)** Tumor vs. normal tissues using TCGA data, **(B)** Tumor vs. normal tissues using TCGA-GTEx data, and **(C)** Tumor vs. paired normal tissues using TCGA data. (*P<0.05, **P<0.01, ***P<0.001; NS, not significant).

The mRNA expression profile of UBTD1 in diverse malignancies was analyzed based on the TCGA database. The TCGA dataset uncovered that UBTD1 mRNA was notably highly expressed in 12 types of malignant tumors, including BRCA, CHOL, COAD, ESCA, GBM, HNSC, LIHC, PCPG, READ, STAD, THCA, and UCEC, whereas its expression was decreased in 7 tumors, namely CESC, KICH, KIRC, KIRP, LUAD, LUSC, and PRAD (P<0.05, **Figure 1A**).

Validation using the integrated TCGA-GTEx dataset highlighted that UBTD1 exhibited higher expression levels across 13 cancer types, including CHOL, GBM, HNSC, LAML, LGG, LIHC, OV, PAAD, PCPG, STAD, THCA, UCEC, and UCS, whereas it was downregulated in 9 cancer types compared with normal tissues (CESC, KICH, KIRC, KIRP, LUAD, LUSC, PRAD, SKCM, and TGCT) (P<0.05, **Figure 1B**).

Furthermore, the examination of paired samples across 23 cancer types implied that UBTD1 mRNA expression was markedly increased in malignant tissues of BRCA, CHOL, COAD, ESCA, HNSC, LIHC, STAD, and THCA compared to their corresponding paracancerous tissues. Conversely, UBTD1 mRNA expression was markedly decreased in KICH, KIRC, KIRP, LUAD, LUSC, and PRAD cancerous tissues as opposed to surrounding non-cancerous tissues (P<0.05, **Figure 1C**).

Immunohistochemical analysis via the HPA database showed detectable UBTD1 protein expression patterns across various types of cancer and the normal tissues that correspond to them, including BRCA, CHOL, COAD, HNSC, KICH, KIRC, KIRP, LIHC, LUAD, LUSC, PRAD, STAD, and THCA (**Supplementary Figure 1**). The HPA database’s concentration of protein validation matched the mRNA expression results. This empirical data at the protein level not only confirmed the biological basis of the above-mentioned transcriptome analysis, but also further strengthened the reliability and robustness of the conclusions of this study.

### Pan-cancer prognostic implications of UBTD1

**Figure 2:**
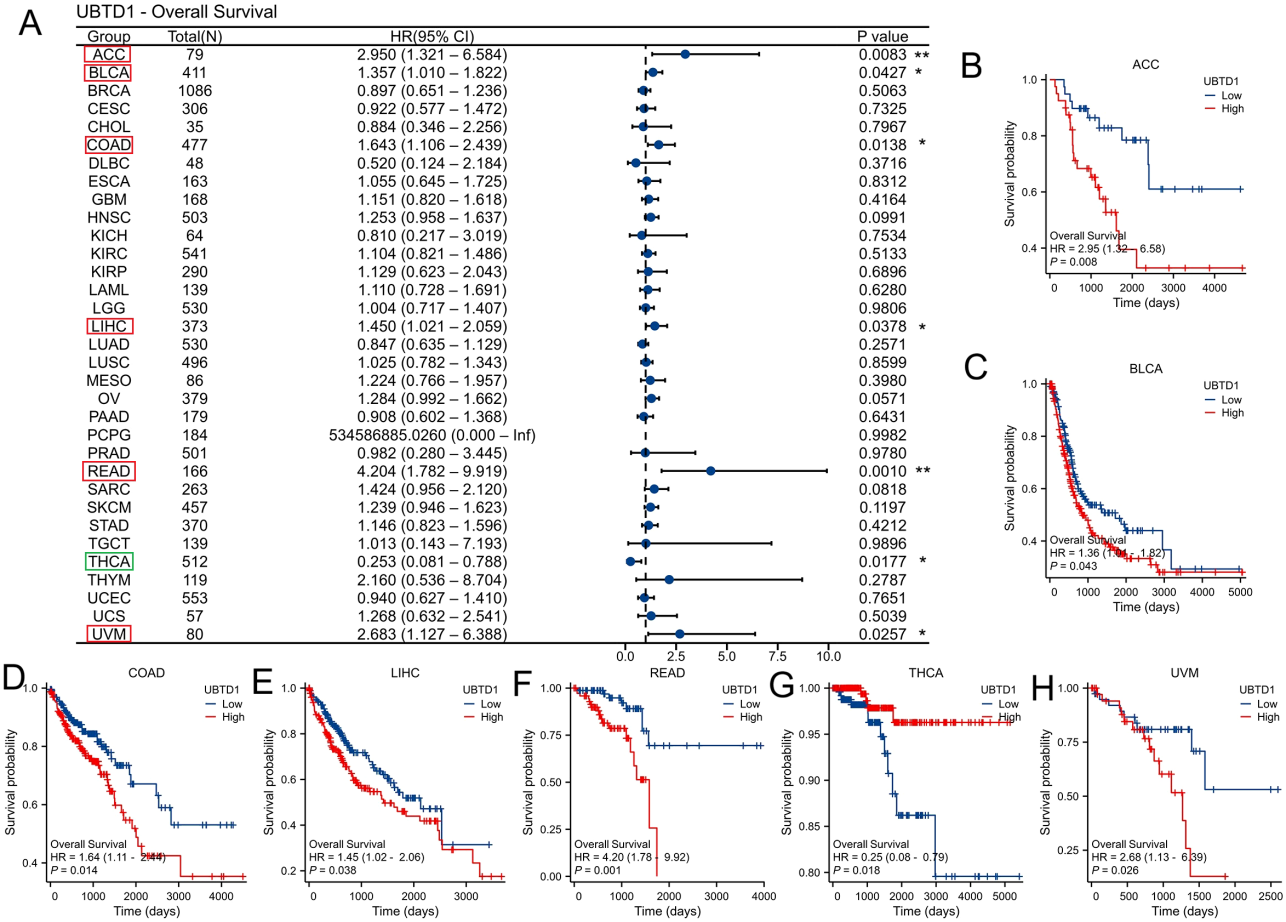
UBTD1 expression in tumor patients and its correlation with prognosis. **(A)** Forest plot illustrating the association between UBTD1 expression and OS. OS analysis stratified by cancer type: ACC **(B)**, BLCA **(C)**, COAD **(D)**, LIHC **(E)**, READ **(F)**, THCA **(G)**, and UVM **(H)**. (*P<0.05, **P<0.01).

This study examined UBTD1’s prognostic significance in pan-cancer using the TCGA database. For OS, reduced expression of UBTD1 was recognized as a negative prognostic factor correlated with decreased OS in patients with THCA (HR = 0.25). In contrast, low UBTD1 expression served as a favorable factor linked to longer OS in patients with ACC (HR = 2.95), BLCA (HR = 1.36), COAD (HR = 1.64), LIHC (HR = 1.45), READ (HR = 4.20), and UVM (HR = 2.68) (P<0.05, **Figure 2**). In summary, high UBTD1 expression is strongly linked to poor prognosis in multiple cancer types, while it is indicative of a favorable prognosis in THCA. Collectively, these data indicate that UBTD1 holds potential as a promising prognostic indicator for various cancers.

### Diagnostic significance of UBTD1 across cancer types

**Figure 3:**
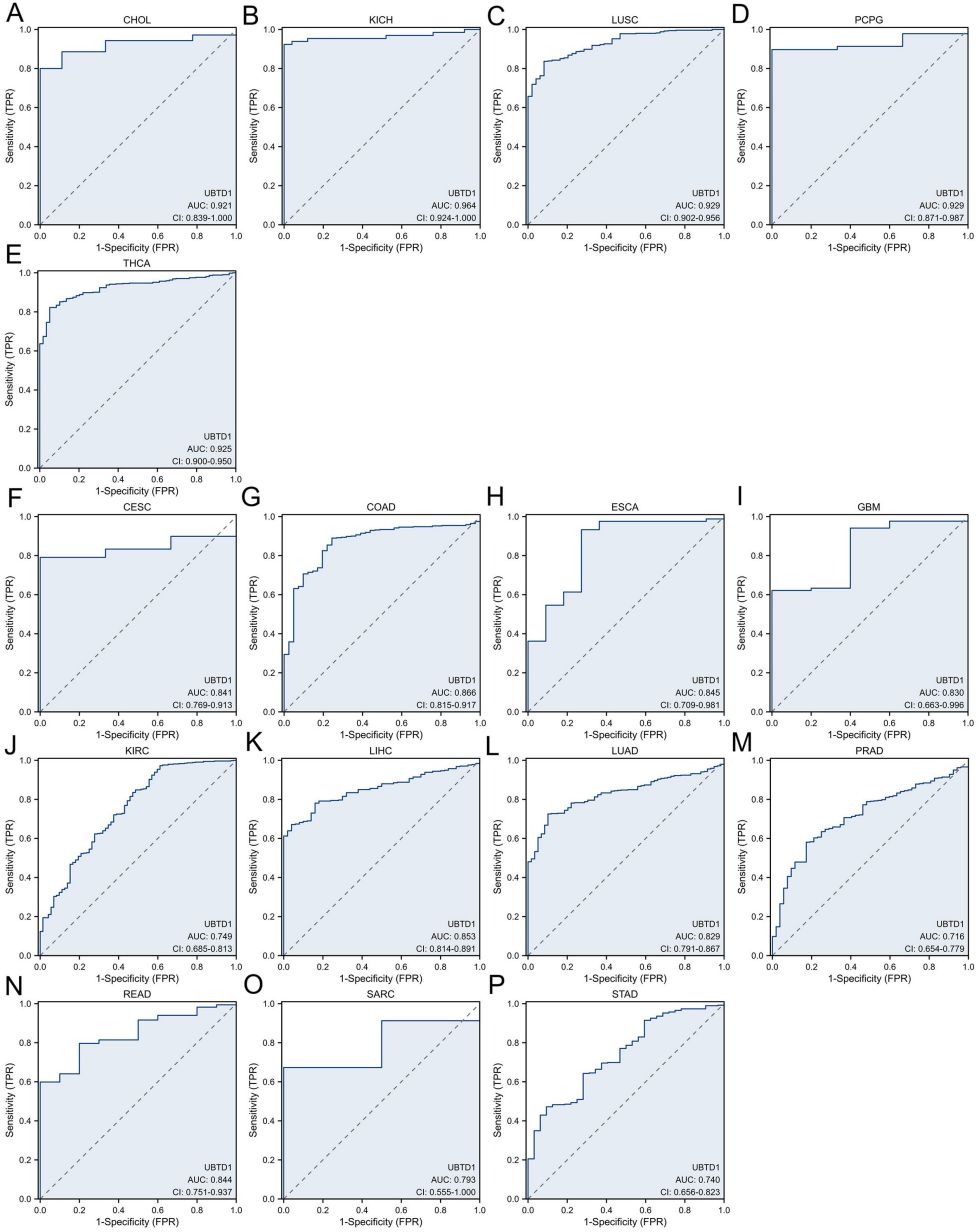
UBTD1 pan-cancer ROC curve analysis. Cancer types with AUC above 0.9: CHOL **(A)**, KICH **(B)**, LUSC **(C)**, PCPG **(D)**, THCA **(E)**. Cancer types with 0.7≤AUC ≤ 0.9: CESC **(F)**, COAD **(G)**, ESCA **(H)**, GBM **(I)**, KIRC **(J)**, LIHC **(K)**, LUAD **(L)**, PRAD **(M)**, READ **(N)**, SARC **(O)**, STAD **(P)**.

To evaluate UBTD1’s diagnostic potential in different cancers, ROC analysis was conducted on the pan-cancer cohort. The results demonstrated that UBTD1 exhibited high diagnostic value in five cancer types, including CHOL (AUC = 0.921), KICH (AUC = 0.964), LUSC (AUC = 0.929), PCPG (AUC = 0.929), and THCA (AUC = 0.925), with all AUC values greater than 0.9 (**Figures 3A–E**). In addition, UBTD1 showed moderate diagnostic value in 11 cancer types: CESC (AUC = 0.841), COAD (AUC = 0.866), ESCA (AUC = 0.845), GBM (AUC = 0.830), KIRC (AUC = 0.749), LIHC (AUC = 0.853), LUAD (AUC = 0.829), PRAD (AUC = 0.716), READ (AUC = 0.844), SARC (AUC = 0.793), and STAD (AUC = 0.740), with AUC values ranging from 0.7 to 0.9 (**Figures 3F–P**). These findings indicate that UBTD1 possesses moderate to substantial diagnostic efficacy across various cancers.

### UBTD1 independently influences cancer prognosis.

This study employed Cox regression analyses to assess factors affecting OS across four cancer types: ACC, LIHC, READ, and THCA (**Supplementary Table 2**). For ACC, pathological T stage (T3&T4, P<0.001, HR = 8.975) and UBTD1 expression level (high, P = 0.039, HR = 2.436) were identified as independent prognostic predictors. For LIHC, pathological stage III/IV (P<0.001, HR = 2.440) and UBTD1 expression level (high, P = 0.046, HR = 1.466) were independent prognostic predictors. For READ, independent prognostic predictors included age (>65 years, P = 0.002, HR = 4.493) and UBTD1 expression level (high, P<0.001, HR = 4.789). For THCA, pathological stage III/IV (P<0.001, HR = 6.908) and UBTD1 expression level (low, P = 0.026, HR = 0.276) were independent prognostic predictors.

**Figure 4:**
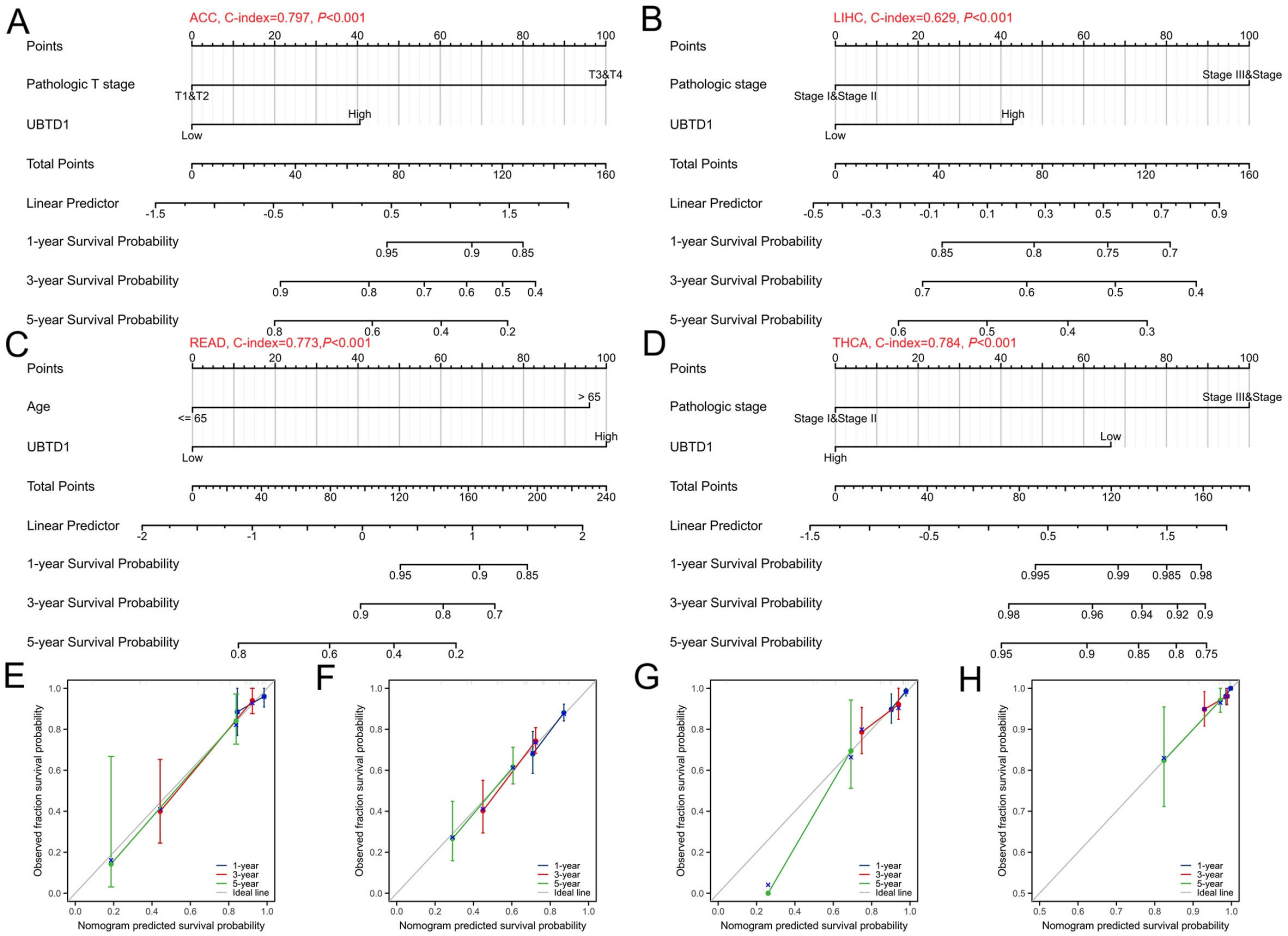
Prognostic tools and calibration models for four cancer types. Prognostic nomograms for patients with ACC **(A)**, LIHC **(B)**, READ **(C)**, and THCA **(D)**. Prognostic calibration curves for patients with ACC **(E)**, LIHC **(F)**, READ **(G)**, and THCA **(H)**.

Those variables with P<0.1 identified by Single-variable Cox regression were adopted to establish forecasting diagrams, and the calibration of the models was subsequently assessed. The C-index for each nomogram was: ACC (0.797), LIHC (0.629), READ (0.773), and THCA (0.784) (**Figures 4A–D**). Calibration curves showed all four cancer types closely aligned with the ideal reference line, indicating UBTD1 is a reliable independent prognostic predictor for these cancers (**Figures 4E–H**).

### Genetic variation profile of UBTD1 in pan-cancer

**Figure 5:**
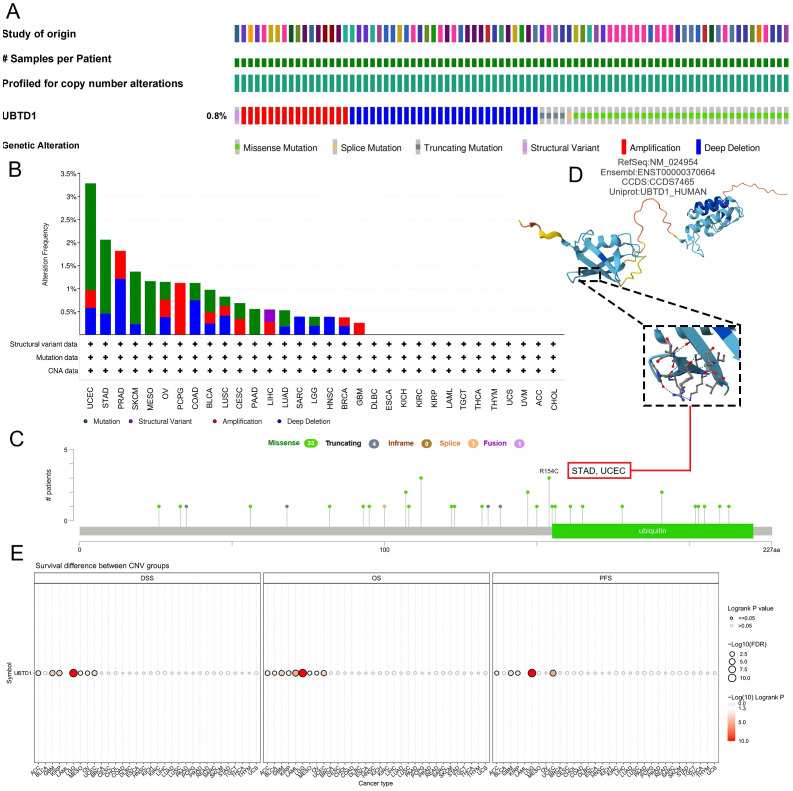
Mutation landscape of UBTD1 in cancers **(A)**. Expression alterations of UBTD1 in different tumors **(B)**. Distribution and localization of mutation sites **(C)**. A three-dimensional structural representation of critical mutations **(D)**. Prognostic impact of UBTD1 copy number variations **(E)**.

Cancers result from various genetic changes, and targeting these key variations is crucial for molecular therapy (24, 25). To explore UBTD1 as a potential molecular therapy target, the present study analyzed 10,967 pan-cancer samples by means of the cBioPortal database, and the genetic alteration profile of UBTD1 was examined across various cancer types. The findings indicated that mutations in the UBTD1 gene were identified in 86 out of 10,967 cases (0.8%), with missense mutations being the most prevalent type (**Figure 5A**). Subsequently, the mutational status of UBTD1 was examined in pan-cancer. Among all cancer types, UCEC (3.21%), STAD (2.05%), PRAD (1.82%), SKCM (1.36%), and MESO (1.15%) displayed the highest prevalence of gene mutations (**Figure 5B**). Notably, R154C was the most common mutation in the UBTD1 domain (**Figure 5C**). Finally, we thoroughly mapped the mutation distribution in both the 3D structure and amino acid sequence of the UBTD1 protein (**Figure 5D**).

Following this, the GSCA database was employed to investigate the associations of UBTD1 mutations with UBTD1 mRNA expression and prognosis in pan-cancer patients. The pie chart depicting CNVs retrieved from this database indicated that the majority of cancers demonstrated heterozygous amplification and deletion, with occasional homozygous amplification in ACC, UCEC, and UCS, as well as sporadic homozygous deletion in DLBC, PRAD, COAD, BLCA, and LUSC (**Supplementary Figure 3B**). Furthermore, a positive correlation was observed between CNV events of UBTD1 and its mRNA expression levels in multiple cancers, including LUSC, OV, SARC, and SKCM (P<0.05; **Supplementary Figure 3C**). Likewise, CNV alterations in UBTD1 were identified as a significant factor contributing to compromised clinical prognosis in patients with ACC, GBM, KIRP, LGG, and UCEC (**Figure 5E**; **Supplementary Figure 3A**). Genetic changes in UBTD1 are common in malignant tumors, impacting cancer prognosis and potentially serving as a target for molecular therapy.

### Assessment of UBTD1 in relation to drug sensitivity

Investigation based on information from the CTRP database indicated a negative association between UBTD1 and drug sensitivity, indicating that elevated UBTD1 expression may be linked to increased drug resistance (**Supplementary Figure 3D**). Subsequent analysis utilizing the GDSC database investigated the relationship between elevated UBTD1 expression and broad-spectrum drug resistance. The findings from our study indicated a bidirectional association between UBTD1 and drug sensitivity in the GDSC database. UBTD1 was inversely associated with sensitivity to most agents, including HDAC inhibitors, CDK inhibitors, and other compounds, while it was positively linked to sensitivity to MEK inhibitors (trametinib, selumetinib, RDEA119), docetaxel, bleomycin, and other compounds. These findings indicate that high UBTD1 expression may enhance sensitivity to certain drugs while reducing responsiveness to others, exhibiting drug-specific effects (**Supplementary Figure 3E**).

### Correlation between UBTD1 expression and methylation

**Figure 6:**
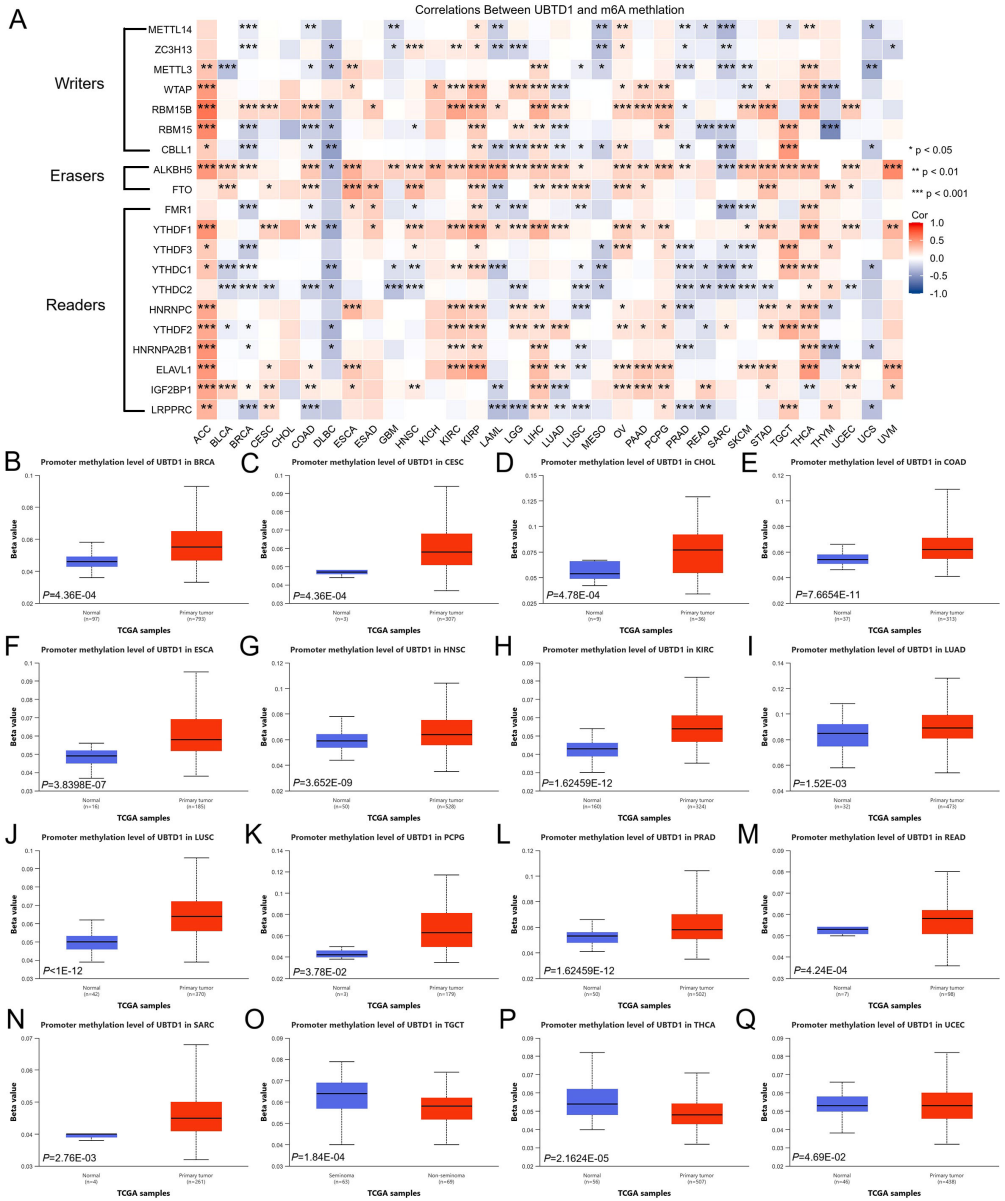
Epigenetic methylation analysis of UBTD1. **(A)** Interaction between UBTD1 and m6A regulators in cancers. Promoter methylation levels in: BRCA **(B)**, CESC **(C)**, CHOL **(D)**, COAD **(E)**, ESCA **(F)**, HNSC **(G)**, KIRC **(H)**, LUAD **(I)**, LUSC **(J)**, PCPG **(K)**, PRAD **(L)**, READ **(M)**, SARC **(N)**, TGCT **(O)**, THCA **(P)**, UCEC **(Q)**. (*P<0.05, **P<0.01, ***P<0.001).

Growing evidence indicates that abnormal DNA methylation is crucial in tumor development (26). To understand the mechanism, we analyzed the correlation between UBTD1 mRNA levels and 24 core m6A methylation regulators across various cancers. Heatmap analysis revealed a positive correlation in most tumors (**Figure 6A**).

A comparative analysis of UBTD1 promoter methylation between normal and tumor specimens was performed using the UALCAN database. It was found through analysis that the UBTD1 promoter was hypermethylated in tumor tissues compared with normal tissues in BRCA (P = 4.36E-04), CESC (P = 4.36E-04), CHOL (P = 4.78E-04), COAD (P = 7.6654E-11), ESCA (P = 3.8398E-07), HNSC (P = 3.652E-09), KIRC (P = 1.62459E-12), LUAD (P = 1.52E-03), LUSC (P<1E-12), PCPG (P = 3.78E-02), PRAD (P = 1.62459E-12), READ (P = 4.24E-04), and SARC (P = 2.76E-03). In contrast, hypomethylation was observed in malignant tissues of TGCT (P = 1.84E-04), THCA (P = 2.1624E-05), and UCEC (P = 4.69E-02) (**Figures 6B–Q**).

### UBTD1 expression linked to tumor immunity

**Figure 7:**
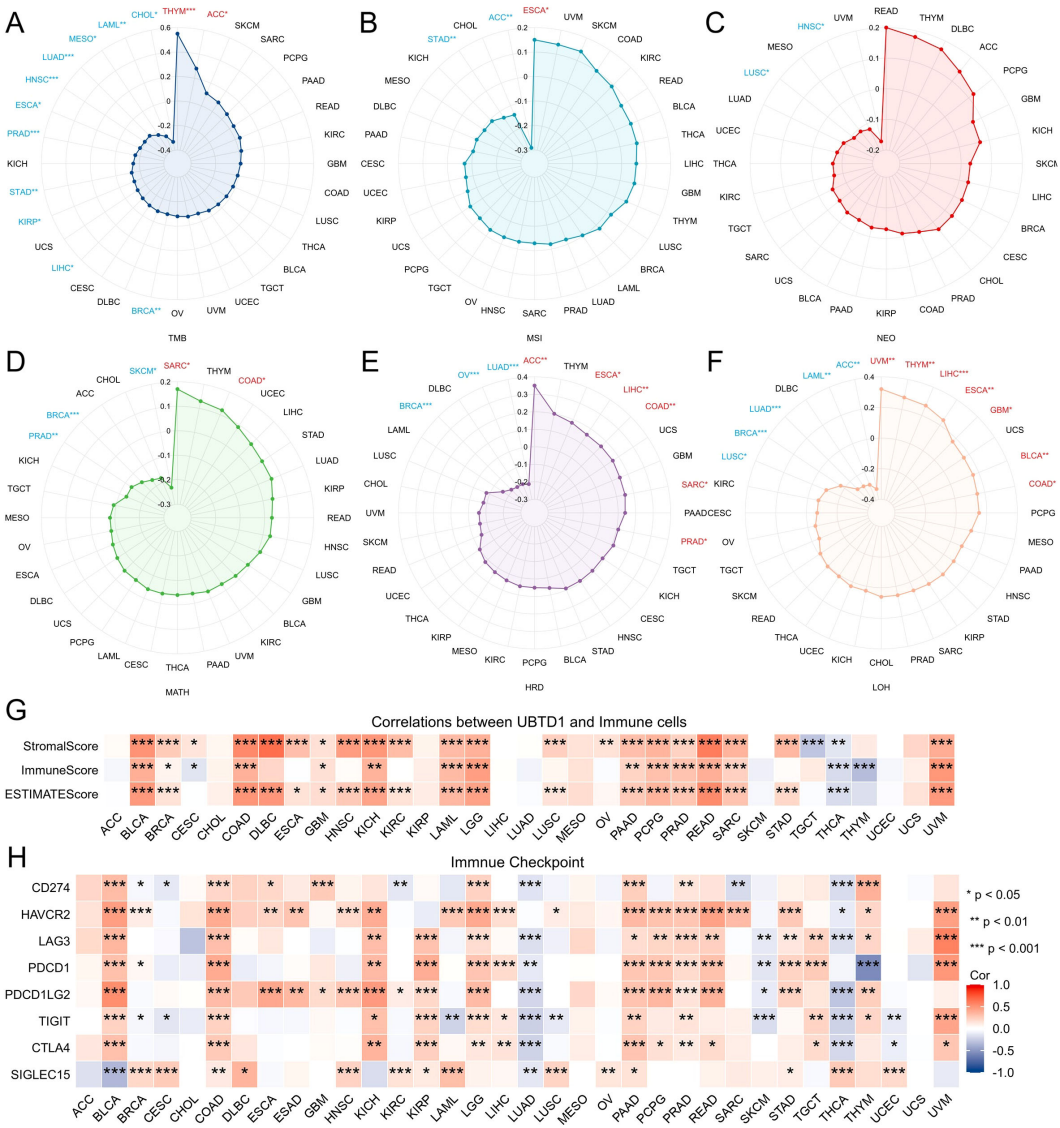
UBTD1 expression levels and immunogenomic features of tumors. **(A–F)** Correlations with TMB, MSI, NEO, MATH, HRD, and LOH. **(G)** Association between UBTD1 and immune infiltration scores. **(H)** Association between UBTD1 and immune checkpoints. (*P<0.05, **P<0.01, ***P<0.001).

Accumulating evidence demonstrates that TMB, MSI, NEO, MATH, HRD, and LOH can function as predictive biomarkers for immunotherapy efficacy. Accordingly, this research thoroughly showed the links between UBTD1 and six genomic biomarkers. Radar chart results demonstrated that UBTD1 expression demonstrated a positive linear correlation with TMB in THYM and ACC, but negatively correlated with TMB in 11 cancer types, including BRCA, LIHC, KIRP, STAD, PRAD, ESCA, HNSC, LUAD, MESO, LAML, and CHOL (P<0.05; **Figure 7A**). There is a notable link between UBTD1 expression and MSI was identified in three cancer types, with a positive correlation in ESCA and negative correlations in STAD and ACC (P<0.05; **Figure 7B**). Similarly, UBTD1 expression demonstrated a negative association with NEO only in LUSC and HNSC (P<0.05; **Figure 7C**). UBTD1 expression showed a positive correlation with MATH in SARC and COAD, but a negative correlation in PRAD, BRCA, and SKCM (P<0.05; **Figure 7D**). For HRD, a positive relationship was observed between UBTD1 expression and ACC, ESCA, LIHC, COAD, SARC, and PRAD, but negatively correlated with BRCA, OV, and LUAD (P<0.05; **Figure 7E**). For LOH, UBTD1 expression was positively correlated with UVM, THYM, LIHC, ESCA, GBM, BLCA, and COAD, and negatively correlated with LUSC, BRCA, LUAD, LAML, and ACC (P<0.05; **Figure 7F**).

Additionally, correlation analyses were performed between UBTD1 mRNA expression levels and stromal score, immune score, and tumor purity score across various cancer types. The data demonstrated that UBTD1 expression was positively associated with these three scores across 13 cancer types, including BLCA, BRCA, COAD, GBM, KICH, LAML, LGG, PAAD, PCPG, PRAD, READ, SARC, and UVM, while showing a negative correlation in THCA (P<0.05; **Figure 7G**).

Further correlation analyses were performed to investigate the connection between immune checkpoint genes and UBTD1 mRNA expression levels. Heatmap results showed that UBTD1 expression was significantly positively correlated with most immune checkpoint genes in BLCA, COAD, KICH, KIRP, LGG, PAAD, and PRAD, conversely, negative correlations were present in LUAD and THCA (P<0.05; **Figure 7H**). Heatmap analysis for immune-activating genes revealed that UBTD1 expression was positively correlated with most immune-activating genes in BLCA, COAD, KIRP, and PAAD (P<0.05; **Supplementary Figure 4A**). Conversely, heatmap analysis of immunosuppressive genes demonstrated a positive association between UBTD1 expression and the majority of immunosuppressive genes in BLCA, COAD, LGG, PCPG, and PRAD, with significant statistical differences (P<0.05; **Supplementary Figure 4B**).

**Figure 8:**
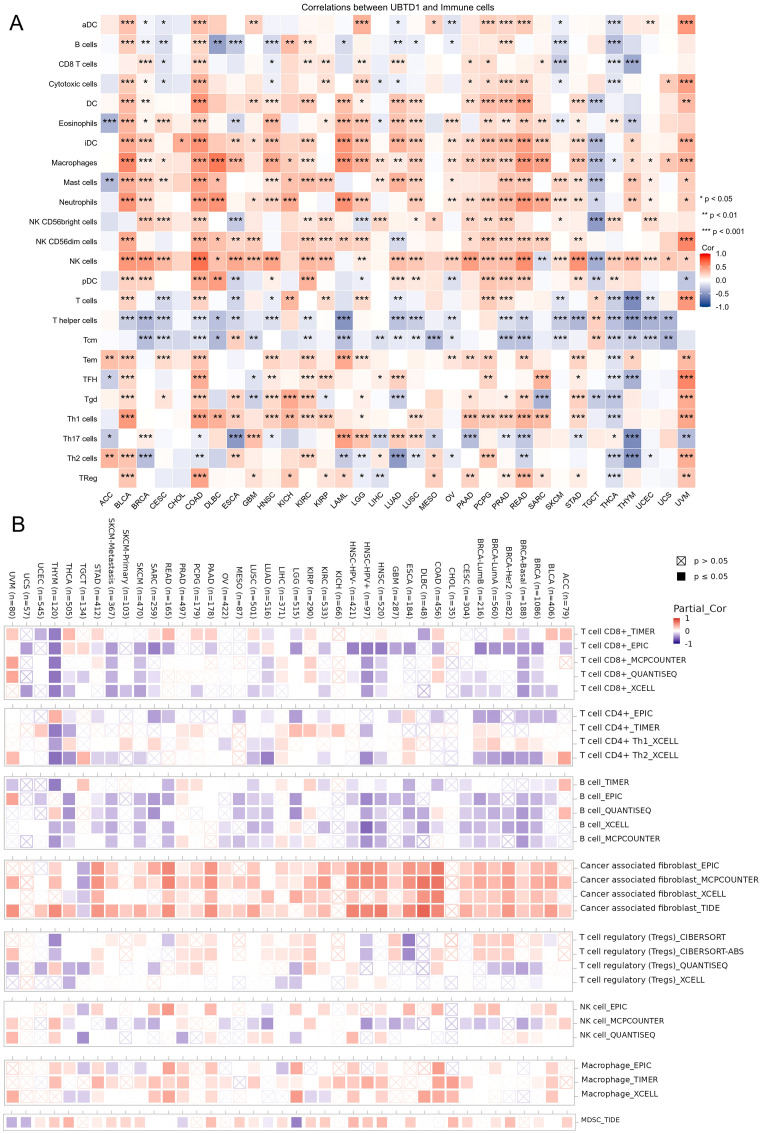
Immune cell infiltration patterns correlated with UBTD1 expression in pan-cancer specimens. **(A)** Analysis using ssGSEA. **(B)** Analysis using TIMER3.0. (*P<0.05, **P<0.01, ***P<0.001).

TIICs, key components of the TME, play a crucial role in cancer therapy. This study employed the single-sample GSEA technique to assess the association between UBTD1 mRNA expression and the infiltration levels of 24 distinct TIICs. Heatmap analysis demonstrated that UBTD1 transcript levels displayed a direct correlation with the infiltration abundance of most TIICs across eight malignancies: BLCA, COAD, HNSC, KIRC, PCPG, PRAD, READ, and UVM. Significant positive correlations were notably identified for dendritic cells (DCs), eosinophils, immature DCs, macrophages, mast cells, neutrophils, natural killer cells, and Th1 cells. Conversely, UBTD1 expression negatively correlated with T helper and central memory T cells in most cancers (**Figure 8A**). Using TIMER 3.0, EPIC, MCP-COUNTER, CIBERSORT, and TIDE, it was found that UBTD1 mRNA expression positively correlated with cancer-associated fibroblast infiltration in the majority of cancers (**Figure 8B**).

### Functional enrichment and PPI network analysis of UBTD1

**Figure 9:**
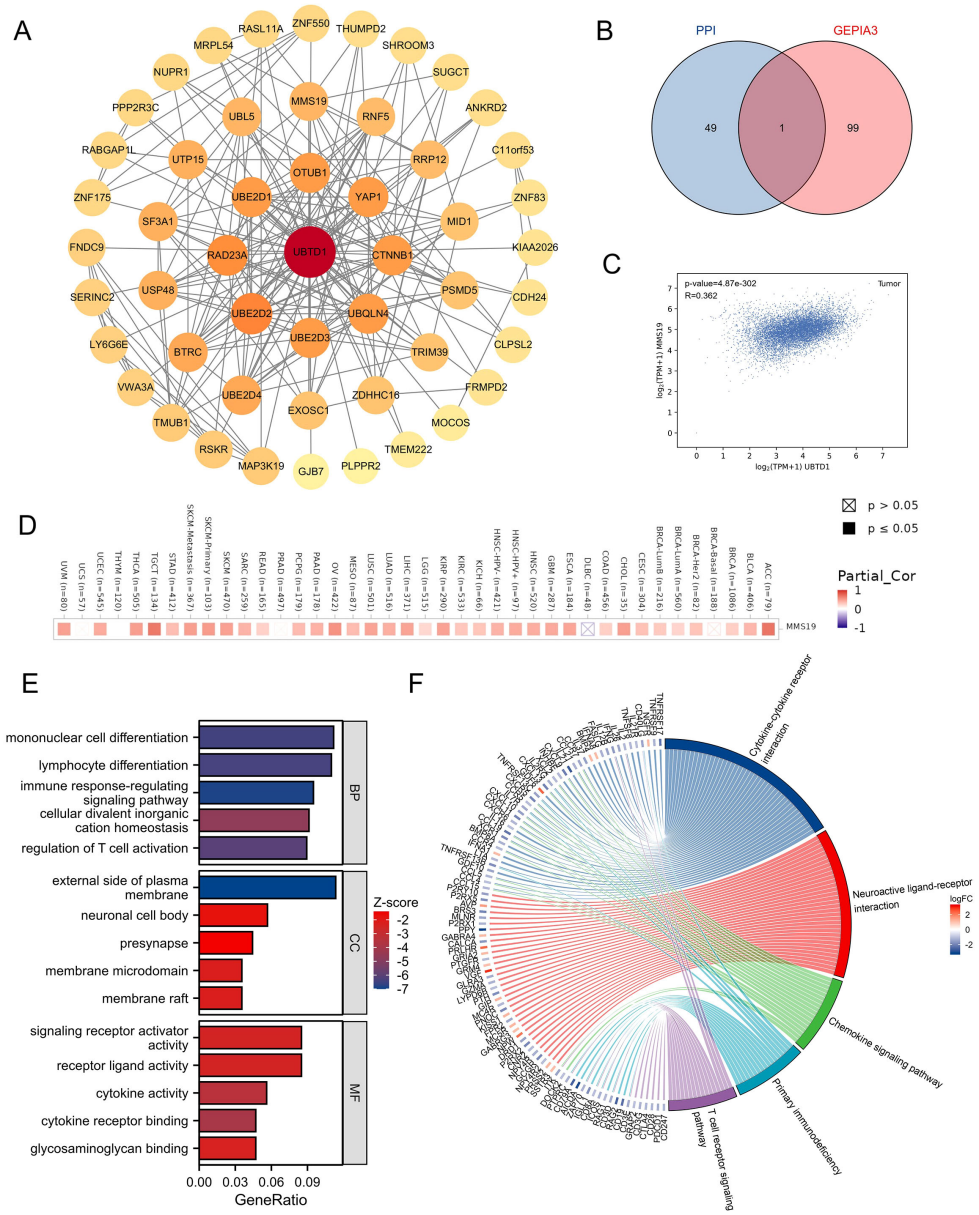
UBTD1 protein-protein interaction network and functional enrichment analysis. **(A)** Network of UBTD1 protein interactions. **(B)** Genes that bind and interact with UBTD1. **(C, D)** Important gene correlations in cancer studies. **(E)** Analyses of GO biological processes, cellular components, and molecular functions. **(F)** Analysis of KEGG pathways.

To investigate the potential pathways involved in UBTD1-mediated carcinogenesis and cancer progression, 50 proteins interacting with UBTD1 were extracted from the STRING database, and a PPI network was constructed (**Figure 9A**). The 100 genes most correlated with UBTD1 were chosen from the GEPIA3 database. A Venn diagram intersection of these two gene sets identified one core molecule, MMS19 (**Figure 9B**). The analysis of the scatter plot revealed a positive correlation between UBTD1 expression and MMS19 expression, with a correlation coefficient of r=0.362 (**Figure 9C**). Additionally, UBTD1 expression demonstrated a positive relationship with MMS19 expression across a large proportion of tumor types examined (**Figure 9D**).

Subsequently, an extensive analysis combining GO and KEGG with log fold change (logFC) enrichment was conducted on UBTD1-binding proteins and associated genes to elucidate the biological functions linked to UBTD1 (**Supplementary Table 3**). This analysis yielded a total of 718 GO terms, comprising 647 terms related to BP, 23 terms pertaining to CC, 48 terms associated with MF, and 28 KEGG pathways. Among tumor-related GO terms, GO-BP analysis revealed that UBTD1 contributed to the differentiation processes of mononuclear cells and lymphocytes and was part of the signaling pathways that regulate immune responses. cellular divalent inorganic cation homeostasis, and regulation of T cell activation. GO-CC analysis showed that UBTD1-related genes were significantly enriched in the external side of the plasma membrane, neuronal cell body, presynapse, membrane raft, and membrane microdomain. In addition, GO-MF analysis suggested that UBTD1-related genes may participate in receptor ligand activity, signaling receptor activator activity, cytokine activity, glycosaminoglycan binding, and cytokine receptor binding (**Figure 9E**). KEGG pathway analysis suggests that UBTD1 may play a role in cytokine-cytokine receptor interactions, neuroactive ligand-receptor interactions, the chemokine signaling pathway, primary immunodeficiency, and the T cell receptor signaling pathway (**Figure 9F**).

In addition, GSEA was performed in five cancer types (ACC, COAD, LIHC, READ, and THCA). The study revealed that there is a positive correlation between genes and UBTD1 expression, and mainly involved in extracellular matrix remodeling, immune regulation, and growth factor signaling pathways. These processes are closely tied to tumor growth and changes in the tumor microenvironment. Genes negatively correlated with UBTD1 expression were mainly enriched in pathways associated with cell cycle progression, DNA/RNA synthetic metabolism, and diverse immune signaling pathways. These findings demonstrate tissue-specific effects across various tumor microenvironments (**Supplementary Figure 2**).

### UBTD1 expression and phenotype regulation in THCA tissues

**Figure 10:**
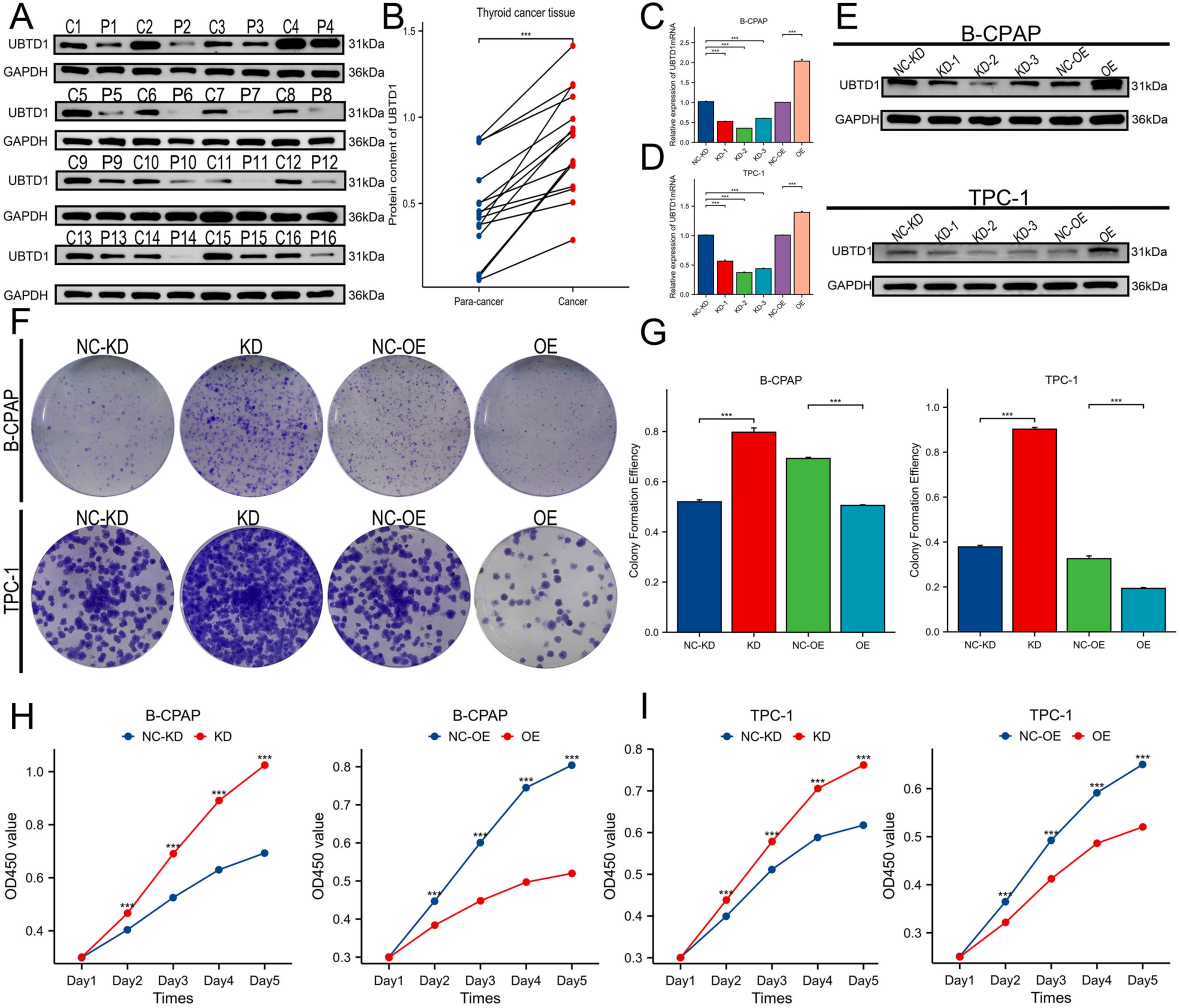
UBTD1 is highly expressed in THCA tissues and inhibits the proliferation of THCA cells. **(A, B)** Western blot analysis verified the expression of UBTD1 protein in THCA tissues and adjacent normal thyroid tissues. **(C-E)** Validation of UBTD1 knockdown and overexpression efficiency in B-CPAP and TPC-1 cell lines. **(F, G)** Colony formation and. **(H-I)** CCK-8 assays demonstrate how varying UBTD1 expression impacts THCA cell proliferation. (***P<0.001).

**Figure 11:**
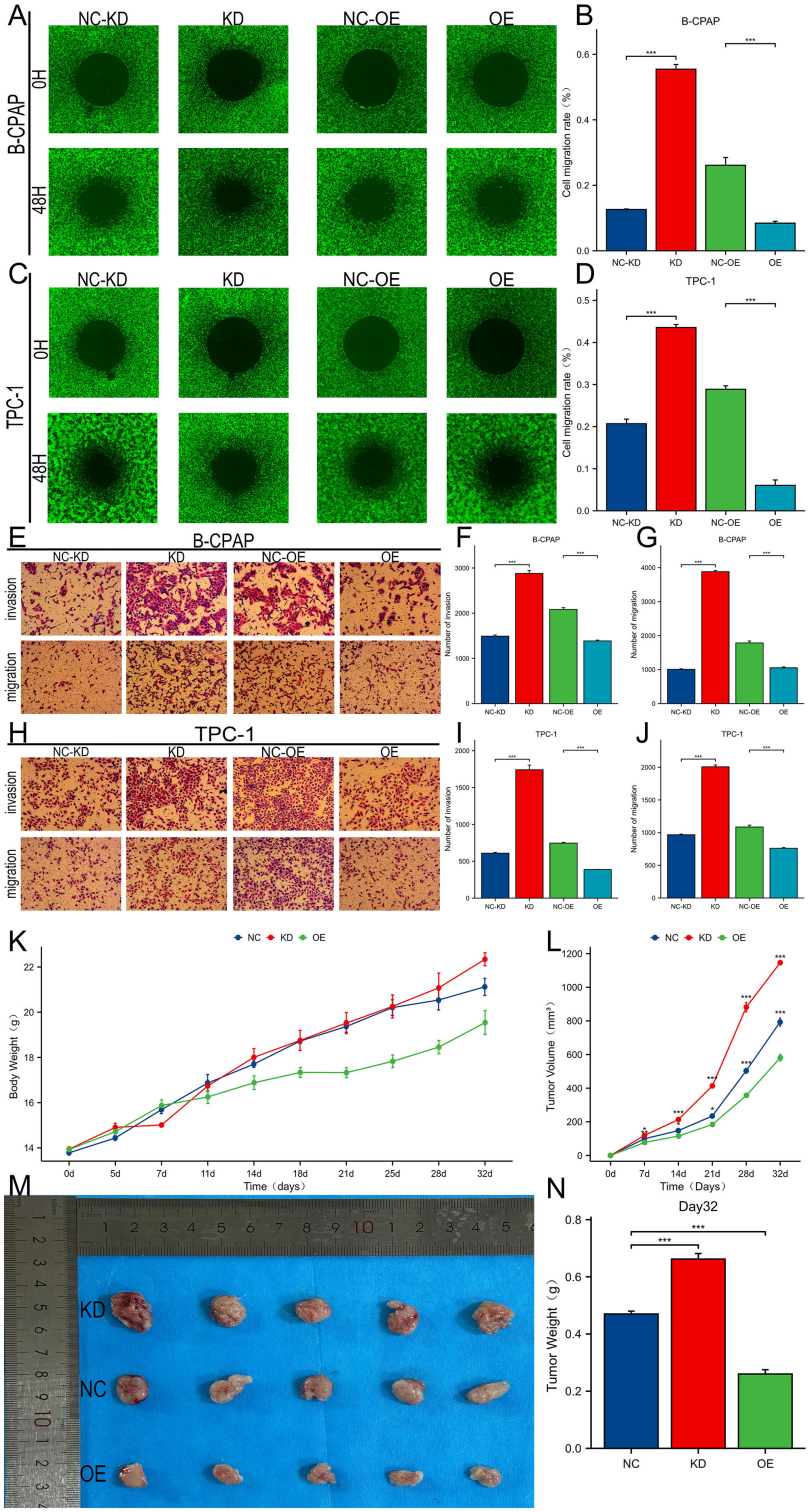
Regulation of UBTD1 in THCA cell lines and mouse models. **(A–D)** Oris assay results on THCA cell migration after UBTD1 knockdown and overexpression. **(E–J)** Transwell assays showing UBTD1’s impact on migration and invasion in B-CPAP and TPC-1 cells. **(K–N)** *In vivo* effects of UBTD1 knockdown and overexpression on tumor growth and body weight in NCG mice. (***P<0.001).

We first used western blot analysis to evaluate UBTD1 expression in THCA tissues and nearby normal tissues. The findings indicated a significantly elevated expression of UBTD1 in THCA samples in comparison to the matched normal tissues (**Figures 10A, B**). To further explore its functional role, we established UBTD1 knockdown (UBTD1-KD) and overexpression (UBTD1-OE) models in B-CPAP and TPC-1 cell lines. qPCR and western blotting confirmed the knockdown and overexpression efficiencies (**Figures 10C–E**). Colony formation assays demonstrated that enforced overexpression of UBTD1 significantly inhibited the proliferative capacity of THCA cells, while its knockdown increased proliferation (**Figures 10F, G**). Consistent results were obtained in CCK-8 assays (**Figures 10H, I**). Regarding cell invasion and migration, Oris cell migration assays revealed that the increased expression of UBTD1 significantly impaired the migratory capacity of B-CPAP and TPC-1 cells. Conversely, the knockdown of UBTD1 was associated with enhanced cell migration, as illustrated in **Figures 11A–D**. Transwell assays revealed that overexpressing UBTD1 significantly reduced cell invasion and migration, whereas knocking down UBTD1 enhanced these processes (**Figures 11E–J**). Furthermore, *in vivo* xenograft experiments in NCG mice revealed that, compared with the control group, UBTD1 overexpression significantly inhibited tumor growth and weight gain, whereas UBTD1 knockdown accelerated tumor progression (**Figures 11K–N**).

## Discussion

Cancer has emerged as a predominant cause of mortality throughout the world. Despite the escalating annual incidence and mortality rates associated with cancer, advancements in tumor diagnosis and treatment have not yet achieved optimal outcomes. Consequently, the investigation of effective strategies for cancer prevention, therapeutic intervention, and prognostic assessment, alongside the exploration of innovative diagnostic markers and therapeutic targets, constitutes an urgent and critical research imperative. Although UBTD1 has been implicated in tumorigenesis, its global biological function across diverse cancer types remains largely unclear. This study used bioinformatics on various datasets to explore UBTD1’s clinical value and potential biological mechanisms in various cancers.

This study conducted differential expression analysis on 33 malignant tumors using both paired and unpaired samples from the TCGA and GTEx databases. UBTD1 expression differed among cancer types, showing increased levels in most tumors. We next assessed the prognostic role of UBTD1 across multiple cancer types. Notably, UBTD1 may exert completely opposite roles in different tumor types. Upregulated UBTD1 expression correlated with worse clinical outcomes in different types of cancer, including ACC, BLCA, COAD, LIHC, READ, and UVM. In contrast, high expression levels of UBTD1 indicated better clinical outcomes in individuals with THCA. Studies conducted previously have found that UBTD1 expression exhibits significantly higher expression in colorectal cancer tissues relative to matched normal adjacent tissues. Moreover, high expression of UBTD1 has been linked to poorer survival outcomes, which is consistent with our present results (27, 28). In addition, UBTD1 may serve as a diagnostic marker for a wide range of malignancies and a standalone prognostic indicator for ACC, LIHC, READ, and THCA, supporting its potential use in cancer treatment and management. Despite its varied roles in different cancers, UBTD1 is generally a crucial prognostic factor. We further evaluated the diagnostic value of UBTD1 in pan-cancer and observed favorable predictive potential in most cancer types. However, more research is needed to assess UBTD1’s potential as a cancer diagnostic biomarker.

One hallmark of cancer is aberrant epigenomic regulation. In this process, dysregulated gene expression reshapes cellular phenotypes, disturbs the homeostatic equilibrium among cells and the TME, and ultimately promotes malignant progression, encompassing tumor initiation, proliferation, invasion, and metastasis (29, 30). According to the present analysis, UBTD1 mutations are widely distributed across diverse cancers and show a positive association with UBTD1 mRNA expression. Furthermore, these mutations correlate accompanied by poor survival outcomes in multiple malignancies. In the majority of cancers, the presence of missense mutations in UBTD1 coincides with elevated UBTD1 mRNA expression and predicts inferior clinical outcomes. We therefore hypothesize that missense mutations of UBTD1 represent a key mechanism contributing to increased UBTD1 mRNA expression, thereby leading to poor prognosis in cancer patients. Therefore, mutations in UBTD1 are probably significant in the development and advancement of tumors. This research is the first to reveal the critical role of UBTD1 mutations across various cancers. However, more experimental research is needed to clarify how UBTD1 mutations affect the onset and progression of cancer.

Tumorigenesis is often linked to abnormal gene expression or genetic changes. Both gene amplification and methylation status can induce aberrant gene expression, thereby accelerating tumor progression (31, 32). Studies indicate that m6A may influence oncogene and tumor suppressor gene expression, impacting tumorigenesis and cancer progression (33). We analyzed the correlations between UBTD1 expression, promoter methylation status, and the expression profiles of m6A methylation−related regulators in diverse cancer types. The findings indicated that the UBTD1 promoter exhibited hypermethylation in the majority of tumor samples. Moreover, UBTD1 expression showed a positive association with m6A methylation−related regulators across most malignant neoplasms, with strong positive correlations in ACC, KIRP, LIHC, OV, and THCA, indicating that UBTD1 may display higher m6A methylation levels in these malignancies. Collectively, UBTD1 methylation modifications are crucial in pan-cancer. Nevertheless, further functional assays are required to elucidate its precise underlying mechanisms.

Tumor immunotherapy strengthens the host’s immune system to enable the recognition and eradication of cancer cells, with ICIs serving as the main therapeutic agents (34, 35). TMB, MSI, and NEO have the potential to be used as predictive markers in cancer therapy. Higher TMB, MSI, and NEO levels are associated with better responses to ICIs and more favorable prognoses (36, 37). In addition, TMB, MSI, NEO, MATH, HRD, and LOH are crucial markers that indicate tumor genomic diversity and have a strong connection to immunotherapy. This study examined the links between UBTD1 expression and TMB, MSI, NEO, MATH, HRD, and LOH, revealing notable specificity to certain cancer types. Our data revealed that UBTD1 exhibited a significant negative correlation with TMB, MATH, HRD, and LOH in BRCA patients, and UBTD1 mRNA was highly expressed in BRCA. This result indicates that elevated UBTD1 expression correlates with lower TMB, MATH, HRD, and LOH scores in BRCA patients, implying that those with high UBTD1 levels may exhibit a poorer response to immunotherapy. However, the role and mechanism of UBTD1 in BRCA require additional exploration in subsequent work.

We next investigated the association between UBTD1 and immune cell infiltration within the TME across diverse cancer types. The findings indicated a positive correlation between UBTD1 mRNA expression and the infiltration levels of various immune cells in COAD, HNSC, PCPG, and READ, including DCs, eosinophils, immature DCs, macrophages, mast cells, neutrophils, natural killer cells, and Th1 cells. These cells primarily participate in antigen presentation, activation of the innate immune system, pro-inflammatory responses, and the initiation of adaptive immunity, thereby constituting the central effector populations responsible for mediating protective immune responses against tumors (38). Combined with the expression profile of UBTD1 in these cancers, we hypothesize that high UBTD1 expression may positively regulate the anti-tumor immune microenvironment by recruiting and promoting the infiltration of the aforementioned immune cells, thereby enhancing local immune surveillance. Jin (39) found UBTD1 to be a key prognostic and predictive gene in colorectal cancer, linked to immune checkpoint regulation and immune cell function, with high expression correlating with increased immune checkpoint levels. Such a conclusion is consistent with our present analysis. As a persistently activated fibroblast population in the TME, CAFs mediate immunosuppression and therapeutic resistance by secreting cytokines, remodeling extracellular matrix, and regulating immune cell infiltration (40). The current investigation showed that elevated UBTD1 mRNA expression coincided with increased CAFs infiltration across most malignancies. We hypothesize that UBTD1 not only regulates immune cell infiltration but may also participate in TME remodeling by modulating stromal components. This study is pioneering in highlighting the significant role of UBTD1 in pan-cancer immunity. However, additional studies are needed to validate these findings.

Moreover, we analyzed the association between UBTD1 and drug sensitivity. Analyses utilizing the CTRP and GDSC databases illustrated a negative association between UBTD1 and susceptibility to multiple chemotherapeutic and targeted drugs. This suggests that elevated UBTD1 expression may be linked to drug resistance in tumor cells. It should be acknowledged that the associations between UBTD1 and drug response observed in this study were all based on retrospective analyses of public databases, and the translational potential of targeting UBTD1 as a predictive biomarker for immunotherapy or chemotherapy remains to be further validated in prospective cohort studies. This study preliminarily revealed a potential correlation between UBTD1 expression and drug resistance, which warrants further exploration in future studies.

To investigate UBTD1’s role in pan-cancer, we conducted GO and KEGG enrichment analyses. GO results showed UBTD1 and its related genes are involved in various biological processes, molecular functions, and cellular components. KEGG analysis indicated UBTD1’s potential involvement in cytokine interactions, neuroactive ligand-receptor interactions, chemokine signaling, primary immunodeficiency, and T cell receptor signaling pathways. To further investigate its possible cellular mechanisms, we selected five cancer types for GSEA. The findings showed that genes positively correlated with UBTD1 expression were mainly enriched in processes related to extracellular matrix remodeling, immune regulation, and growth factor signal transduction. This further supported our hypothesis that UBTD1 not only regulates immune cell infiltration but may also participate in tumor microenvironment construction by remodeling stromal components, thereby affecting tumorigenesis and cancer progression.

Although UBTD1 showed an overall risk trend at the pan-cancer level, preliminary bioinformatics analysis showed that UBTD1 had a unique expression and prognostic pattern in THCA: The expression level of UBTD1 in THCA tumor tissues was significantly higher than that in normal thyroid tissues, and the prognosis of the high expression group was significantly better than that of the low expression group. This paradoxical “high express-good prognosis” association, which is extremely rare in most solid tumors, suggests that UBTD1 may play a distinct role in THCA. Therefore, we selected THCA as the validation object precisely to explore the molecular mechanisms underlying this tissue-specific functional switch in depth and to evaluate the potential of UBTD1 as a THCA-specific prognostic marker. It should be pointed out that the vast majority of THCA, especially papillary carcinoma, grows slowly, has an indolent course, and has an excellent prognosis, which is one of the best prognosis of solid tumors. In this context of already good overall prognosis, the pattern of association between the expression of many genes and prognosis may differ from that seen in other more aggressive malignancies. In this specific context, we speculate that the elevated baseline expression of UBTD1 in THCA may represent an ‘aborted cellular defense mechanism’ or a compensatory response attempting to restrain tumor progression. Alternatively, this upregulation could be intrinsically linked to the maintenance of cellular differentiation, a hallmark of indolent thyroid cancers, rather than driving malignancy. Our experimental observations provide phenotypic evidence consistent with this hypothesis. Both *in vitro* and *in vivo* investigations demonstrated that enforced overexpression of UBTD1 significantly inhibited the proliferation, migration, and invasion of THCA cells, whereas its knockdown produced the opposite effects. These findings confirm the tumor-suppressive function of UBTD1 in THCA, supporting the notion that its upregulation contributes to a less aggressive phenotype. In fact, this “high expression and good prognosis” correlation is not unique to UBTD1 in indolent THCA. For instance, in papillary thyroid carcinoma, high expression of IGF2BP1 predicts a better five-year survival rate and significantly inhibits the proliferation, invasion, and migration of thyroid cancer cells (41). However, whether UBTD1 specifically exerts its protective effect in thyroid cancer by activating the cellular senescence program or promoting differentiation remains to be further verified by *in vitro* and *in vivo* experiments. In summary, our findings shed new light on the function of UBTD1, which could support the advancement of cancer treatment strategies.

## Conclusion

Through the application of bioinformatic analyses, this study constitutes the first comprehensive systematic exploration of the expression profile, prognostic and diagnostic value, epigenetic characteristics, methylation status, immune features, and pathway enrichment of UBTD1 across multiple cancer types. In cancer research, UBTD1 emerged as a groundbreaking prognostic biomarker and a promising therapeutic target. We confirmed its expression and regulatory role in THCA through lab and animal studies. The research improves understanding into UBTD1’s role in cancer development and provides valuable clues for designing innovative immunotherapy approaches.

## Data Availability

The original contributions presented in the study are included in the article/**Supplementary Material**. Further inquiries can be directed to the corresponding author.

## Glossary

ACC: Adrenocortical Carcinoma
BLCA: Bladder Urothelial Carcinoma
BRCA: Breast Cancer
BP: Biological processes
CESC: Cervical squamous cell carcinoma and endocervical adenocarcinoma
CHOL: Cholangiocarcinoma
CNV: Copy Number Variants
COAD: Colon adenocarcinoma
CC: Cellular components
DLBC: Lymphoid Neoplasm Diffuse Large B-cell Lymphoma
DSS: Disease-specific Survival
ESCA: Esophageal carcinoma
GBM: Glioblastoma multiforme
HNSC: Head and neck squamous cell carcinoma
HRD: Homologous recombination deficiency
IHC: Immunohistochemistry
KICH: Kidney chromophobe cell carcinoma
KIRC: Kidney renal clear cell carcinoma
KIRP: Kidney renal papillary cell carcinoma
LAML: Acute Myeloid Leukemia
LGG: Brain Lower Grade Glioma
LIHC: Liver hepatocellular carcinoma
LUAD: Lung adenocarcinoma
LUSC: Lung squamous cell carcinoma
LOH: Loss of heterozygosity
MSI: Microsatellite Instability
MATH: Mutant-allele tumor heterogeneity
MF: Molecular functions
NEO: Neoantigen
OS: Overall Survival
OV: Ovarian serous cystadenocarcinoma
PAAD: Pancreatic adenocarcinoma
PFS: Progression-free Survival
PPI: Protein-protein Interaction
PRAD: Prostate adenocarcinoma
READ: Rectum adenocarcinoma
SKCM: Skin Cutaneous Melanoma
STAD: Stomach adenocarcinoma
TGCT: Testicular Germ Cell Tumors
THYM: Thymoma
TMB: Tumor Mutational Burden
TME: Tumor Microenvironment
UCEC: Uterine corpus endometrial carcinoma
UCS: Uterine Carcinosarcoma
WB: Western blotting
