# Supplementary material for: UBTD1: a prognostic and immune biomarker validated in thyroid cancer and pan-cancer analysis
Source: Front Oncol. 2026 Jul 8;16:1850798. doi: 10.3389/fonc.2026.1850798 (PMC13388116; doi:10.3389/fonc.2026.1850798)
Supplement: Supplementary file 1 [file DataSheet1.docx]

**Supplementary materials for**

**UBTD1: A Prognostic and Immune Biomarker Validated in Thyroid Cancer and Pan-Cancer Analysis.**

**Baoguo Xu^1^, Yue Zhang^2^, Xia Yu^3^, Chenming Guo^1*^**

^1^The First Affiliated Hospital of Xinjiang Medical University, Urumqi 830054, China;

^2^Department of Oncology, Bayingol Mongol Autonomous Prefecture People's Hospital, Korla 841000, China;

^3^Department of Dermatology, Xinjiang Uygur Autonomous Region Children's Hospital, Urumqi 830000, China;

^*^Corresponding author

^*^Correspondence: Chenming Guo, [gcm_xjmu@yeah.net](mailto:gcm_xjmu@yeah.net).

Additional material for this article can be found in the Supplementary Graphics and Table Legend module.


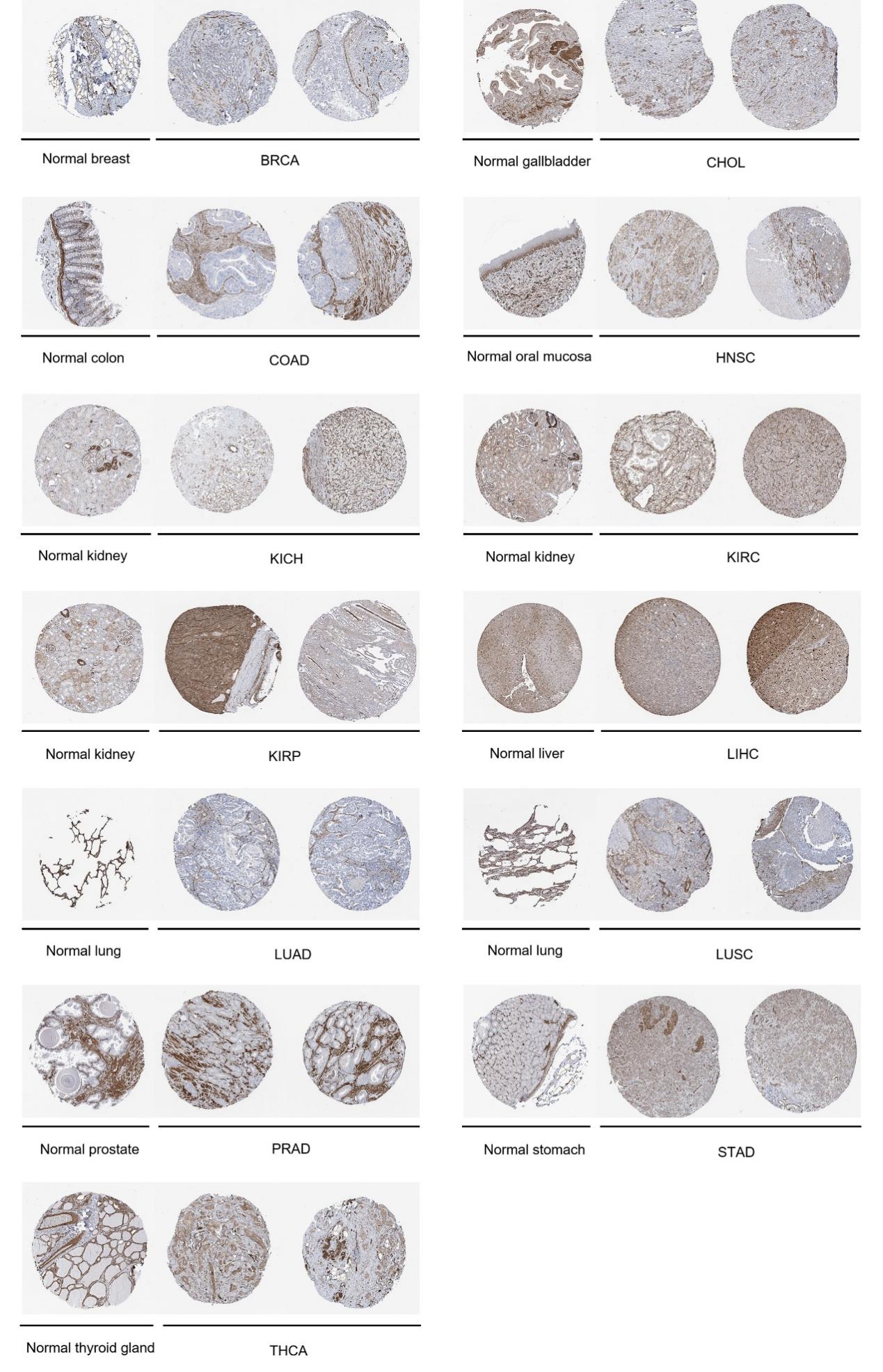


**Supplementary Figure S1.** Immunohistochemical Staining of 13 Normal Tissues and Tumor Tissues from the HPA Database.

**
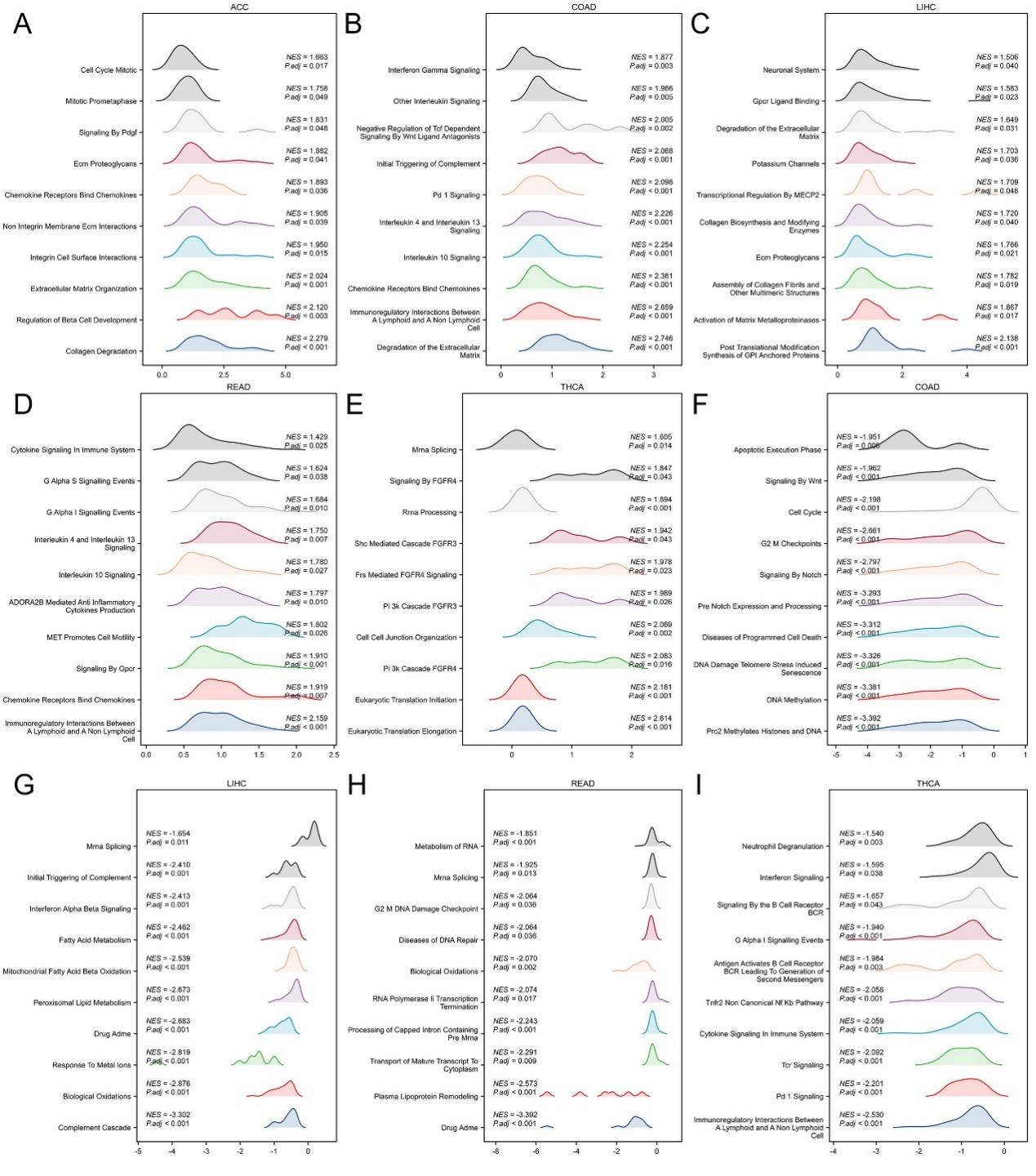
**

**Supplementary Figure S2.** GSEA functional enrichment analysis of UBTD1 in five types of cancer. In ACC **(A)**, COAD **(B)**, LIHC **(C)**, READ **(D)**, THCA **(E)**, the top ten pathways were positively correlated with UBTD1 expression. In COAD **(F)**, LIHC **(G)**, READ **(H)**, and THCA **(I)**, the top ten pathways were negatively correlated with UBTD1 expression.


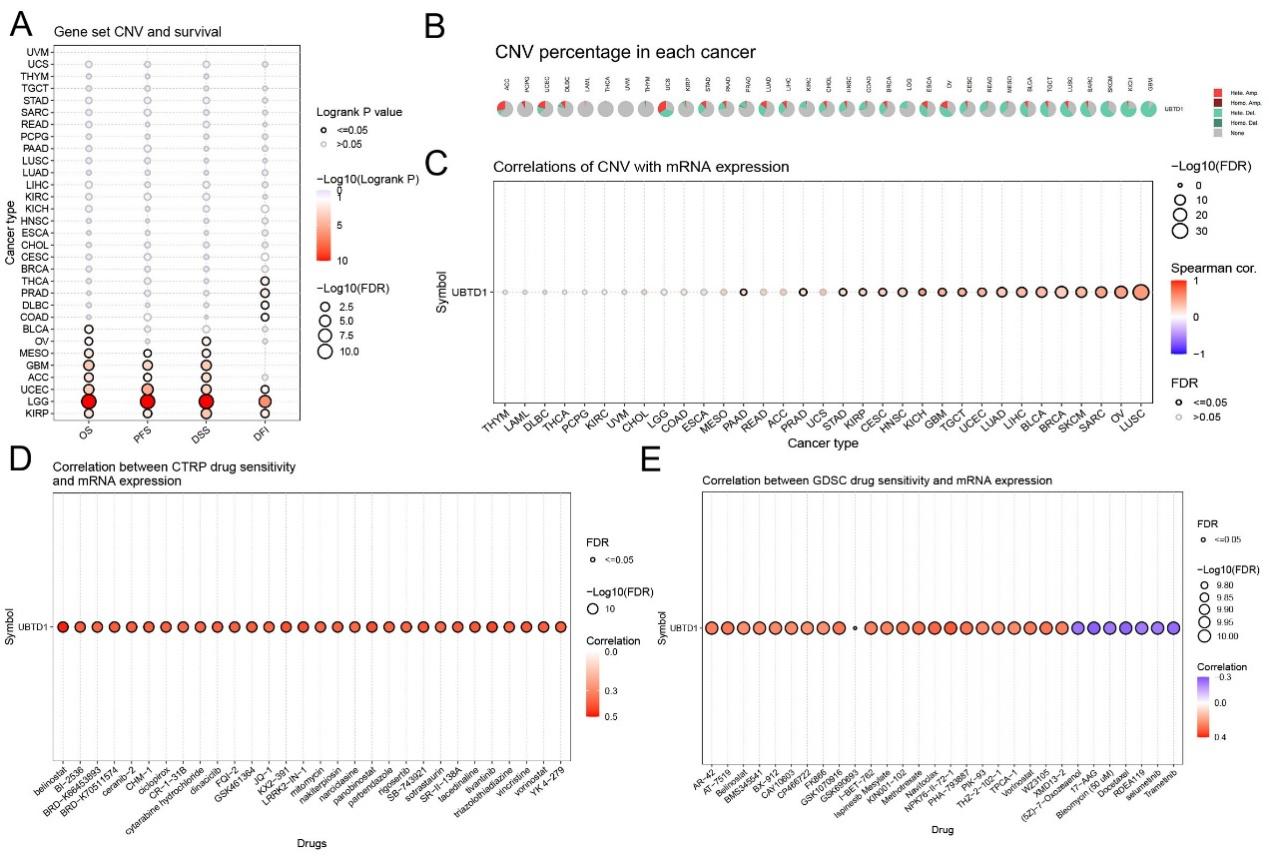


**Supplementary Figure S3.** Mutation analysis and drug sensitivity analysis of UBTD1. **(A)** Gene set CNV and survival of UBTD1. **(B)** Percentage of UBTD1 CNVs in each cancer. **(C)** Correlation between UBTD1 expression and CNVs. **(D)** Correlation between CTRP drug sensitivity and UBTD1 expression. **(E)** Correlation between GDSC drug sensitivity and UBTD1 expression.


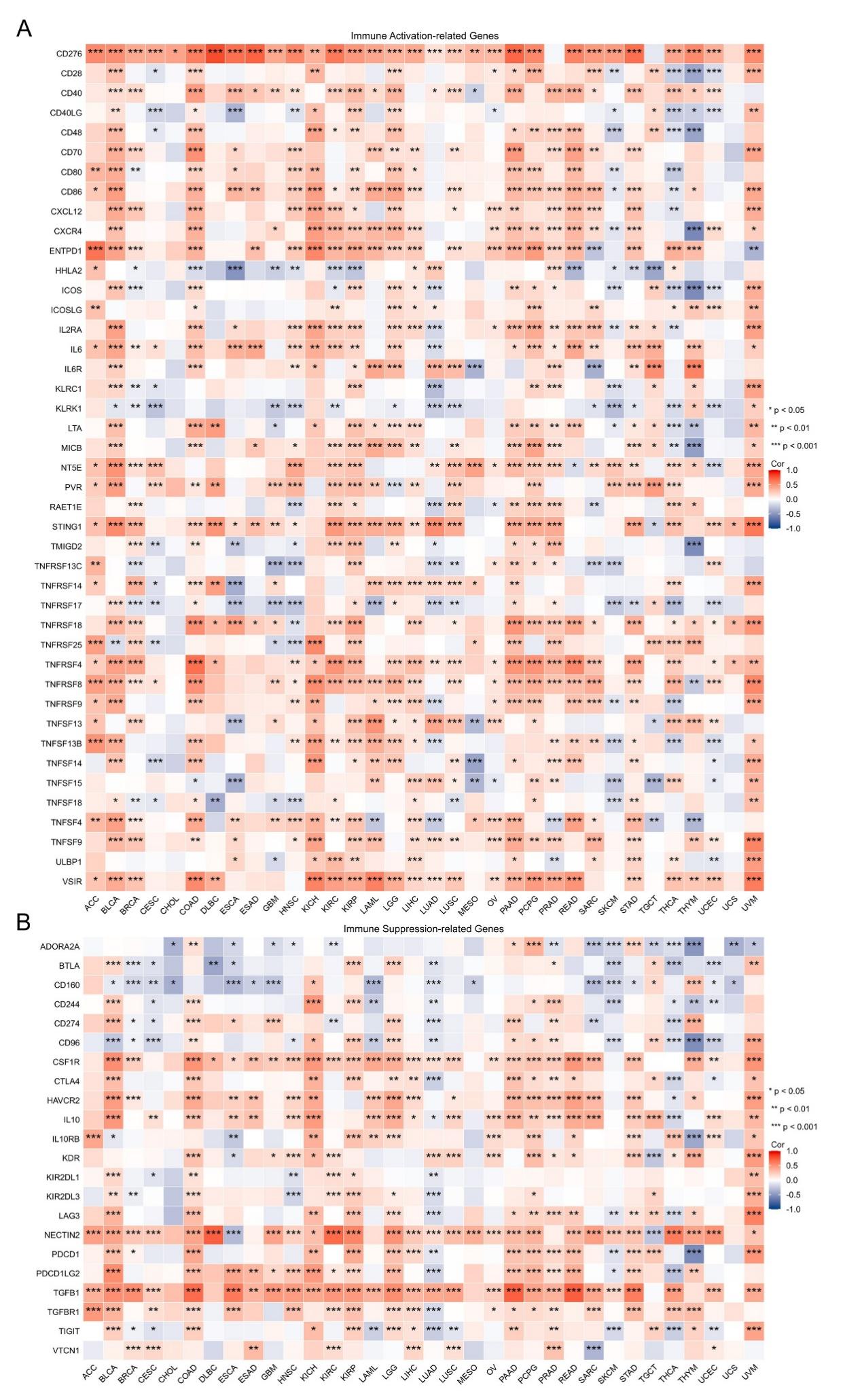


**Supplementary Figure S4.** The relationship between UBTD1 and immune genes. **(A)**Immune activation-related genes. **(B)** Immune suppression-related genes.

**Supplementary Table S1.** UBTD1 shRNA sequences and primer sequences.

| Name | Targeting sequence (5'→3') |
| --- | --- |
| sh-UBTD1-1 | CCTCTGTGAATGCTACGATGA |
| sh-UBTD1-2 | CTGCCCATCTACTGCCTGTCA |
| sh-UBTD1-3 | ACCCTCTGTGAATGCTACGAT |
| UBTD1-OE-F | ATGATATTCCCCAAACAATACCCAATTATAAACTTTACC |
| UBTD1-OE-R | TTATGATGATGATGATGATGAAACTGTGACGATGGTGGAGGTGC |
| UBTD1-F | GAGCTCTCGATGTCGGGCTG |
| UBTD1-R | CTTCTTCAAGGGCTCATTGCG |
| Beta-actin-F | GGCTGTATTCCCCTCCATCG |
| Beta-actin-R | CCAGTTGGTAACAATGCCATGT |
| GAPDH-F | GTCTCCTCTGACTTCAACAGCG |
| GAPDH-R | ACCACCCTGTTGCTGTAGCCAA |

**Supplementary Table S2.** Results of univariate and multivariate Cox analysis of clinical parameters in ACC **(A)**, LIHC **(B)**, READ **(C)**, THCA **(D)**.

A. ACC

| Characteristics | Total(N) | Univariate analysis | |  | Multivariate analysis | |
| --- | --- | --- | --- | --- | --- | --- |
|  |  | Hazard ratio (95% CI) | P value |  | Hazard ratio (95% CI) | P value |
| Pathologic T stage | 77 |  |  |  |  |  |
| T1&T2 | 51 | Reference |  |  | Reference |  |
| T3&T4 | 26 | 10.286 (3.976 - 26.608) | **< 0.001** |  | 8.975 (3.443 - 23.393) | **< 0.001** |
| UBTD1 | 79 |  |  |  |  |  |
| Low | 39 | Reference |  |  | Reference |  |
| High | 40 | 2.950 (1.321 - 6.584) | **0.008** |  | 2.436 (1.045 - 5.680) | **0.039** |

B. LIHC

| Characteristics | Total(N) | Univariate analysis | |  | Multivariate analysis | |
| --- | --- | --- | --- | --- | --- | --- |
|  |  | Hazard ratio (95% CI) | P value |  | Hazard ratio (95% CI) | P value |
| Pathologic stage | 349 |  |  |  |  |  |
| Stage I&Stage II | 259 | Reference |  |  | Reference |  |
| Stage III&Stage IV | 90 | 2.504 (1.727 - 3.631) | **< 0.001** |  | 2.440 (1.682 - 3.540) | **< 0.001** |
| UBTD1 | 373 |  |  |  |  |  |
| Low | 186 | Reference |  |  | Reference |  |
| High | 187 | 1.450 (1.021 - 2.059) | **0.038** |  | 1.466 (1.007 - 2.135) | **0.046** |

C. READ

| Characteristics | Total(N) | Univariate analysis | |  | Multivariate analysis | |
| --- | --- | --- | --- | --- | --- | --- |
|  |  | Hazard ratio (95% CI) | P value |  | Hazard ratio (95% CI) | P value |
| Age | 166 |  |  |  |  |  |
| <= 65 | 82 | Reference |  |  | Reference |  |
| > 65 | 84 | 3.843 (1.535 - 9.622) | **0.004** |  | 4.493 (1.772 - 11.391) | **0.002** |
| UBTD1 | 166 |  |  |  |  |  |
| Low | 83 | Reference |  |  | Reference |  |
| High | 83 | 4.204 (1.782 - 9.919) | **0.001** |  | 4.789 (2.028 - 11.305) | **< 0.001** |

D. THCA

| Characteristics | Total(N) | Univariate analysis | |  | Multivariate analysis | |
| --- | --- | --- | --- | --- | --- | --- |
|  |  | Hazard ratio (95% CI) | P value |  | Hazard ratio (95% CI) | P value |
| Pathologic stage | 510 |  |  |  |  |  |
| Stage I&Stage II | 340 | Reference |  |  | Reference |  |
| Stage III&Stage IV | 170 | 7.263 (2.337 - 22.573) | **< 0.001** |  | 6.908 (2.224 - 21.458) | **< 0.001** |
| UBTD1 | 512 |  |  |  |  |  |
| Low | 256 | Reference |  |  | Reference |  |
| High | 256 | 0.253 (0.081 - 0.788) | **0.018** |  | 0.276 (0.089 - 0.858) | **0.026** |

**Supplementary Table S3.** GO terms and KEGG pathways enriched in the analysis.
